# An Inter‐Cooperative Biohybrid Platform to Enable Tumor Ablation and Immune Activation

**DOI:** 10.1002/advs.202207194

**Published:** 2023-06-14

**Authors:** Feiyu Li, Qiang Chu, Zefeng Hu, Zijie Lu, Chao Fang, Gaorong Han, Yike Fu, Xiang Li

**Affiliations:** ^1^ State Key Laboratory of Silicon and Advanced Semiconductor Materials School of Materials Science and Engineering Zhejiang University Hangzhou 310027 China; ^2^ ZJU‐Hangzhou Global Science and Technology Innovation Center Zhejiang University Hangzhou 311215 China; ^3^ Tea Research Institute College of Agriculture and Biotechnology Zhejiang University Hangzhou 310058 China

**Keywords:** Baker's yeast, biohybrid materials, layered double hydroxides, metabolism, tumor therapy

## Abstract

A biohybrid therapeutic system, consisting of responsive materials and living microorganisms with inter‐cooperative effects, is designed and investigated for tumor treatment. In this biohybrid system, S_2_O_3_^2−^‐intercalated CoFe layered double hydroxides (LDH) are integrated at the surface of Baker's yeasts. Under the tumor microenvironment, functional interactions between yeast and LDH are effectively triggered, resulting in S_2_O_3_^2−^ release, H_2_S production, and in‐situ generation of highly catalytic agents. Meanwhile, the degradation of LDH in the tumor microenvironment induces the exposure of the surface antigen of yeast, leading to effective immune activation at the tumor site. By virtue of the inter‐cooperative phenomena, this biohybrid system exhibits significant efficacy in tumor ablation and strong inhibition of recurrence. This study has potentially offered an alternative concept by utilizing the metabolism of living microorganisms and materials in exploring effective tumor therapeutics.

## Graphical abstract

S_2_O_3_^2−^ intercalated CoFe layered double hydroxides are integrated with living yeast to modify the sulfur metabolism of yeast for tumor treatment. The two components of this biohybrid platform enable unique inter‐cooperative functionalities, leading to significant efficacy in tumor ablation and immune activation. This platform offers an alternative concept to utilize the metabolism of microorganisms for therapeutic biohybrid materials design.

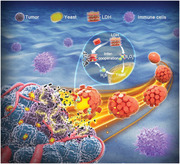

## 1 Introduction

The vast diversity of microorganisms provides considerable potential in the exploration of tumor therapeutics. Unique characteristics of microbes have been utilized for tumor treatment such as targeted delivery, immune stimulation, and glucose consumption.^[^ ^1^, ^2^, ^3^, ^4^, ^5^ ^]^ Despite extensive studies reported in recent years, the understanding of microorganisms‐involved tumor therapeutics, fomented by so‐called biohybrid materials, remains in a rather infant state. A common strategy is that microbes serve as effective carriers and immune stimulators in targeted delivery, photo‐thermal therapy, or chemodynamic therapy.^[^ ^6^, ^7^, ^8^, ^9^ ^]^ Diverse metabolic pathways endow living microorganisms with the production of antitumoral substance but are hardly explored due to the mismatched conditions between tumor microenvironment (TME) and threshold for the occurrence of certain metabolisms (e.g., temperature, acidity, resource). The current protocols, utilizing the metabolism of microbes to consume specific ingredients in TME,^[^ ^10^, ^11^, ^12^ ^]^ suffer low efficacy due to limited metabolites or ingredients. With these insights, materials with tailored functionalities, explored for nanomedicine purposes, may potentially provide the necessary substance or modulate the TME to maintain the sustained metabolisms of native microbes. In addition, the integration of functional materials may also empower microbes with escape properties from immune surveillance during systematic circulation. On the other hand, the metabolites of microbes may also potentially promote the functionalities of materials at the disease sites. The smart integration of TME‐responsive nanomaterials and living microbes may inspire a type of “inter‐cooperative” therapeutic platform to tackle major diseases with promoted efficacy, that is, cancer.

*Saccharomyces cerevisiae*, also called “Baker's yeast” or “Brewer's yeast”, is one of the fungi closely associated with human life.^[^ ^13^, ^14^ ^]^ It is known as probiotics and can transform sulfur resources (e.g., thiosulfates, sulfates, thiols) into H_2_S through sulfur metabolisms, causing rotten‐egg odor in the wine brewing industry.^[^ ^15^, ^16^, ^17^ ^]^ Unlike its unfavorable role played in wine brewing, H_2_S has been extensively recognized as an effective gas molecule in tumor inhibition due to its unique signaling and reductive properties. The reduction properties of H_2_S may also exhibit the potential to promote the catalytic properties of some ions such as Fe^3+^. Nevertheless, compared with ≈160–700 mg L^−1^ of sulfate in grape juice, the blood concentrations of thiosulfate and sulfate in the human system are as low as ≈11.3 and ≈5–10 mg L^−1^, respectively. As a result, the sulfur metabolism of yeast is significantly restrained in an in vivo environment, and its effectiveness in tumor inhibition is limited by a considerable magnitude.^[^ ^18^, ^19^ ^]^ This difference limits the sulfur metabolism of yeast in an in vivo environment and restains our hypothesis of directly applying the sulfur metabolism of yeast in tumor inhibition.

Layered double hydroxides present the microstructure consisting of host hydroxide layers and interlayered space, showing the potential in compensating for the lack of sulfur resources in biological systems. This type of 2D material has received extensive attention for its alterable metal ions, high positive charge density in hydroxide layers, and abilities of anion loading in interlayers.^[^ ^20^, ^21^ ^]^ Sulfur‐contained anions can be feasibly loaded on interlamination of layered double hydroxides to facilitate yeast metabolism. In addition, the selected metal ions in hydroxide layers may present a catalytic effect, which has been utilized for the purpose of tumor inhibition. The unique electropositive layers match the electronegative yeast cytomembranes, paving the way for the self‐assembly of layered double hydroxides at the surface of yeasts.^[^ ^22^ ^]^ It is noteworthy that, due to the nature of hydroxides, layered double hydroxides present typical acid‐responsive degradation, promoting the H_2_S productivity of yeast at tumor sites.^[^ ^23^, ^24^, ^25^ ^]^

**Figure 1:**
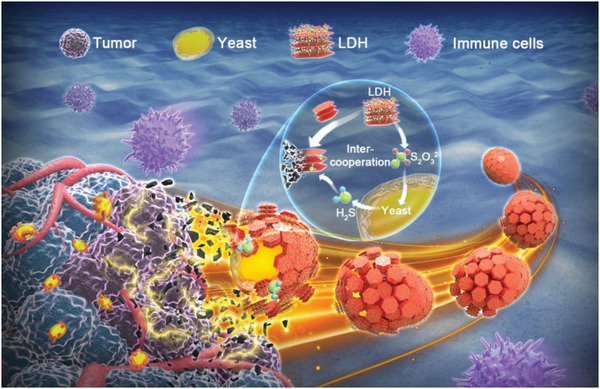
Schematic illustration of inter‐cooperative Y@LDH biohybrid platform for tumor ablation and immune reactivation.

Inspired by the facts, nanoparticles of thiosulfate‐intercalated CoFe layered double hydroxides (LDH) are investigated, and integrated with Baker's yeast (Yeast@CoFe LDH‐S_2_O_3_, denoted as Y@LDH) to construct an inter‐cooperative biohybrid platform for tumor treatment. In this system, two counterparts facilitate each other to trigger a series of unique phenomena after reaching tumoral sites in a targeted manner, including S_2_O_3_^2−^ release, H_2_S production, and in situ generation of catalytic agents. Due to the responsive degradation to TME, LDH with low immunogenicity effectively shield antigens of yeast surface, and activate the antigen due to exposure at the tumor site, as illustrated in **Figure** **1**. The unique inter‐cooperative effects and localized immune stimulation appear to enable us to utilize living microorganisms functionalized by nanomaterials for tumor treatment with promoted effectiveness.

## 2 Results and Discussion

### 2.1 Characteristics of the Y@LDH Platform

**Figure 2:**
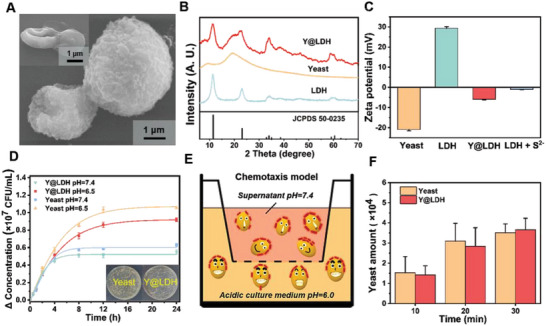
Design and preparation of Y@LDH. A) SEM image of Y@LDH (inset: unmodified yeast). B) XRD spectrum of Y@LDH, yeast, and LDH. C) Zeta potential of yeast, LDH, Y@LDH, and LDH+S^2−^. D) Growth curves of yeast in culture medium with different pH values before and after LDH modification (inset: the same amount of yeast and Y@LDH directly coating on NYDB agar culture medium). E) Schematic illustration of the acid model to evaluate the acid‐induced chemotaxis of Y@LDH. F) The migration quantities of yeast and Y@LDH to the bottom chambers in the acid‐induced chemotaxis model. Data are presented as the mean ± SD. CFU, colony‐forming unit. A. U., arbitrary unit.

To construct the Y@LDH platform, we first purified commercial “Angel instant dry yeast” to obtain native yeast. LDH, another component of the platform, was synthesized using a facile coprecipitation method modified from the preparation of MgAl LDH‐SO_3_^2−^ and NiFe LDH‐S_2_O_3_^2−^.^[^ ^26^, ^27^ ^]^ The detection of sulfur element in EDS mapping indicates the intercalation of S_2_O_3_^2−^ into LDH (Figure S1, Supporting Information). Subsequently, the Y@LDH platform was acquired by mixing the two components in ultrapure water as observed by scanning electron microscopy (SEM), transmission electron microscopy (TEM), and light microscopy (**Figure** **2**A and Figure S2, Supporting Information). The efficient compounding is attributed to a 100‐fold difference in size between the two components (Figure S3, Supporting Information). Powder X‐ray diffraction (XRD) analysis further confirms the successful anchoring of LDH on yeasts (Figure 2B). The LDH is well‐crystalized (Figure S4, Supporting Information), giving LDH a high positive *ζ*‐potential (29.3 mV) opposite to yeast (−20.9 mV), which facilitates the surface assembly via electrostatic interactions (Figure 2C). The anchoring stability of LDH on yeast was investigated by immersion tests in both water and phosphate buffer solution (PBS). After 6 h, most content of LDH remains on the surface of yeast, while clear morphologic change occurs on yeast during the procedure of sample preparation (Figure S5A,B, Supporting Information). Meanwhile, LDH appears a significant reduction of *ζ*‐potential to almost neutral (−0.95 mV) after adding S^2−^ (Figure 2C). This phenomenon implies that LDH may tend to detach from the surface of yeast in the microenvironment containing H_2_S, as observed (Figure S5C, Supporting Information). Furthermore, by mixing 10^7^ colony‐forming units (CFU) yeast with different quantities of LDH, we observed that the corresponding *ζ*‐potentials of Y@LDH varied linearly with the added weight of LDH. To ensure the negative *ζ*‐potential of the hybrids for stable LDH integration, we chose and determined the ratio of 10^7^ CFU yeast to 0.5 mg of LDH for subsequent experiments (Figure S6, Supporting Information). Another intriguing finding was that LDH anchoring significantly improved yeast viability in mouse whole blood (Figure S7, Supporting Information). Given that whole blood contains functional host factors and white blood cells capable of eliminating microorganisms, LDH anchoring can thus be considered a protective measure for yeast.

The active metabolism of yeast is a key element for triggering and maintaining the inter‐cooperative process in Y@LDH. To evaluate the activity of yeast after LDH modification, Y@LDH and yeast were cultured in both normal and acidic culture mediums. No significant difference was observed in both growing curves and NYDB plate colony models (Figure 2D), indicating that the activity of yeast was barely changed after the integration of LDH at its surface. In addition, the acid‐induced chemotaxis of baker's yeast was investigated via the assay using an 8 µm Transwell system (Figure S7, Supporting Information). For the native yeast, the migration quantity towards pH = 6.0 culture medium could reach 5‐folds to the quantity towards pH = 7.4 culture medium. Y@LDH presented similar migration quantities compared with native yeast, implying the acid‐targeting ability of Y@LDH (Figure 2F,G). Considering the lack of active motility in yeast cells, the migration difference could be attributed to the higher possibility of dramatic morphological change of yeast for budding when they approach acidic environments.^[^ ^28^ ^]^ This morphological change leads to a higher possibility of yeast catching and getting through pores in Transwell membranes, and the consequence of that can be regarded as the acid‐targeting ability. Furthermore, the phenomenon that most yeasts leaked to pH = 6.5 are under germination supports this explanation compared with that most yeasts leaked to pH = 7.4 culture medium were in a single‐cell state under light microscope observation (Figure S8C,D, Supporting Information). Overall, Y@LDH was successfully prepared while the intrinsic activity of native yeast was retained.

### 2.2 Responsive Inter‐Cooperative Effect

**Figure 3:**
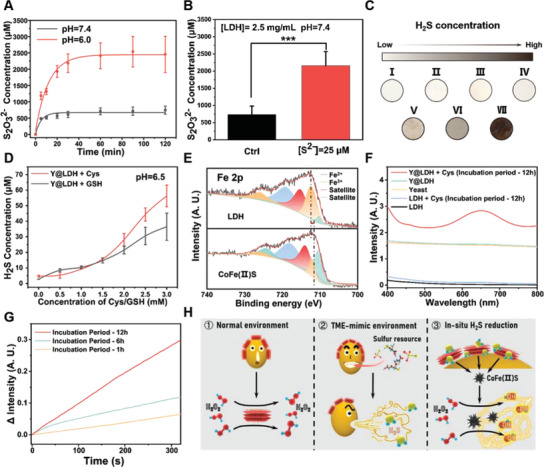
TME‐mimic environment‐triggered inter‐cooperation of Y@LDH. A) S_2_O_3_^2−^ release profiles of LDH at pH 7.4 and 6.0. B) S_2_O_3_^2−^ release amount after additionally adding Na_2_S at pH 7.4. C) H_2_S production in different groups (I: Ctrl, II: LDH‐S_2_O_3_^2−^, III: Cys IV: yeast, V: yeast + LDH‐S_2_O_3_^2−^, VI: Yeast + Cys, VII: Yeast + LDH‐S_2_O_3_^2−^ + Cys). D) H_2_S release profiles of Y@LDH in different concentrations of Cys/GSH. E) Fe 2p XPS spectra of CoFe LDH‐S_2_O_3_ with or without S^2−^. F) UV–vis absorbance spectra with the addition of H_2_O_2_ and various catalyzers. G) Time‐course absorbance at 652 nm after different incubation periods. H) Schematic illustration of TME‐mimic environment‐triggered H_2_S release and enhancement of catalytic activity. Data are presented as the mean ± SD. A. U., arbitrary unit. Statistical significance was analyzed via the two‐tailed Student's *t‐*test (***p < 0.001, **p < 0.01, and *p < 0.05).

The loading capacity of anions in LDH materials is directly related to the excess positive charge of the hydroxide layer in their microstructure. The hydroxide nature of LDH makes it susceptible to disruption by acids, which can degrade the structure and release interlayer ions. Given the acidic microenvironment of tumor tissues, we investigated the effect of acidity on LDH by conducting a standard titration process to measure the release of S_2_O_3_^2−^ from LDH (Figure S8, Supporting Information). The introduction of acid was found to accelerate the release of S_2_O_3_^2−^ (**Figure** **3**A). Additionally, we found that S^2−^, which is a metabolite of yeast sulfur metabolism, also accelerates the release of S_2_O_3_^2−^ (Figure 3B). Furthermore, TEM analysis revealed that both acid and S^2−^ destroyed the LDH nanosheet structure (Figure S10A, Supporting Information), and the color change of LDH after adding S^2−^ confirmed their reaction (Figure S10B, Supporting Information), which will be further discussed later in this section. The S_2_O_3_^2−^ release content from LDH in acid environments for a period of 120 min reached ≈0.982 mmol g^−1^, indicating that more than 11 wt% of S_2_O_3_^2−^ was loaded in LDH during the synthesis. Considering S_2_O_3_^2−^ is an effective resource for the sulfur metabolism of yeast to produce H_2_S, these results suggest that layered double hydroxides with high anionic loading capacity are ideal candidates to deliver metabolic resources for microorganisms.

H_2_S production of yeast was examined with a setup designed by our groups (Figure S11, Supporting Information). Yeast produces more H_2_S in an acidic environment compared with that in a neutral environment (Figure S12A, Supporting Information). The increased H_2_S concentration in the testing bottle verified the promoted induction of H_2_S in an aqueous solution containing higher concentrations of cysteine (Cys) and glutathione (GSH), (Figure S12B, Supporting Information), which are also highly expressed in tumor tissues. After the surface integration of LDH, the metabolism of yeast was facilitated due to the supply of additional S_2_O_3_^2−^ as sulfur resource in an acidic environment, and thus more content of H_2_S was produced (Figure 3C,D). Interestingly, an initial oxygen supply to yeast was found to be in favor of higher H_2_S production, similar to how experienced winegrowers in the wine industry give yeast an initial dose of oxygen to achieve the initial yeast proliferation and increase the efficiency of wine‐making (Figure S12C, Supporting Information). This result implies that when Y@LDH is circulated along the bloodstream into hypoxic tumor tissues, the initial O_2_ supply in the bloodstream could benefit yeast to produce H_2_S at the tumor site.

It is noteworthy that, compared with the as‐prepared layered double hydroxide particles reported earlier,^[^ ^29^ ^]^ the LDH in this study exhibited limited catalytic effect even in pH = 5.0 solution (Figure S13, Supporting Information). This could be attributed to the fact that LDH synthesized in this work was not a mono‐layered microstructure. As observed by the atomic force microscope (AFM), the thickness of LDH was 7.13 nm (Figure S14, Supporting Information), indicating that one LDH particle mainly consists of several hydroxide layers and thus active catalytic sites are lacking. However, when exposed to H_2_S, LDH transformed into a new amorphous phase as shown in Figure S15, Supporting Information, similar to the previous study.^[^ ^30^ ^]^ As shown in Equations (1) and (2), the reactions that occurred involved the dissociation of H_2_S produced by yeast as well as the immediate reaction of S^2−^ with metal hydroxides.

(1) $$H_{2}S\to {\mathrm{HS}}^{-}+H^{+}$$

(2) $$\begin{array}{ccc} & & {\left[Co_{3}\mathrm{Fe}{\left(\mathrm{OH}\right)}_{8}\right]}^{+}{\left(S_{2}O_{3}^{2-}\right)}_{0.5}+4.5HS^{-}\\ & & \;\to Co_{3}\mathrm{Fe}\left(\mathrm{III}\right)S_{4.5}+3.5OH^{-}+0.5S_{2}O_{3}^{2-}++4.5H_{2}O \end{array}$$

Since Fe(III)S is widely known to be unstable in neutral and acidic conditions, S^2−^ further reduces Fe(III) into Fe(II) as shown in Equation (3).

(3) $$2Co_{3}\mathrm{Fe}\left(\mathrm{III}\right)S_{4.5}\to 2Co_{3}\mathrm{Fe}\left(\mathrm{II}\right)S_{4}+S$$

The Fe 2p X‐ray photoelectron spectroscopy (XPS) results of reaction products supported this process (Figure 3E). After the reactions, iron underwent a clear valence transfer from +3 to +2, endowing the newly formed sulfides (CoFe(II)S) with significantly enhanced catalytic activities (Figure S16A, Supporting Information). Similar to most sulfides, the catalytic activity of CoFe(II)S is pH‐dependent because of the decomposition of CoFe(II)S and exposure of Fe^2+^ at low pH (Figure S16B, Supporting Information). The velocity of ROS production of CoFe(II)S, quantified against different concentrations of H_2_O_2_, exhibited typical Michaelis–Menten kinetics (Figure S17A,B, Supporting Information). The maximum velocity (*V_max_*) and Michaelis–Menten constant (*K_M_*) reached 4.46 × 10^−7^ M s^−1^ and 58 × 10^−3^ M, respectively. The production of hydroxyl radicals (·OH) and catalytic enhancement were also identified via electron spin resonance (ESR) spectroscopy (Figure S18, Supporting Information). To evaluate whether this enhancement can occur in Y@LDH, Y@LDH was incubated in a culture medium with Cys, the extracellular thiol formed in a GSH cycle, and then centrifuged for a catalytic test. It became clear that the catalytic activity of Y@LDH was promoted with the extension of the incubation period (Figure 3F,G). These findings confirmed the occurrence of inter‐cooperative effects in the TME.

Overall, the unique inter‐cooperation in the Y@LDH platform was demonstrated to favor the production of H_2_S and enhance catalytic activity in a TME‐mimicking environment (Figure 3H). The acid‐induced release of S_2_O_3_^2−^ promotes the sulfur metabolism of yeast. In return, yeast‐produced H_2_S reacts with LDH, enabling the agitated enhancement in the catalytic property of LDH and the further release of S_2_O_3_^2−^.

### 2.3 In Vitro TME‐Triggered Antitumor Effect

**Figure 4:**
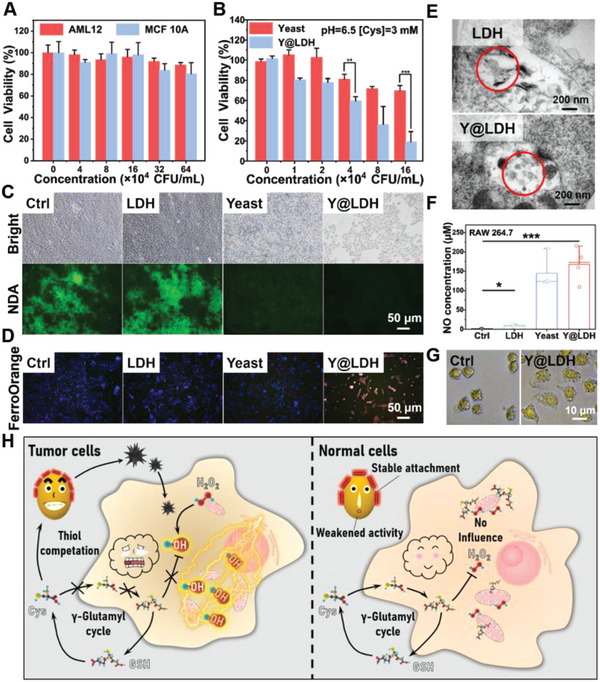
In vitro antitumor properties. A) Cytocompatibility of Y@LDH to AML 12 cells and MCF 10A cells (*n* = 6). B) Viability of murine mammary carcinoma cell 4T1 cultured with different concentrations of yeast and Y@LDH under TME‐simulated culture medium (*n* = 6). C) Light field and Fluorescent images of 4T1 cells stained with NDA to detect the intracellular thiol. D) FerroOrange staining of 4T1 cells for detecting intracellular Fe^2+^. E) Bio‐TEM images of 4T1 cells cultured with LDH and Y@LDH in TME simulated culture medium to illustrate the endocytosis of inter‐cooperative production. F) NO concentration in RAW 264.7 macrophage culture medium after different treatments. G) Morphology change of RAW 264.7 macrophages cultured with Y@LDH. H) Schematic illustration of the inter‐cooperative process for tumor therapy. Data are presented as the mean ± SD. CFU, colony‐forming unit. Statistical significance was analyzed via the two‐tailed Student's *t‐*test (***p < 0.001, **p < 0.01, and *p < 0.05).

In a normal culture medium, Y@LDH did not exhibit any clear negative effects on murine hepatocyte cell line AML12 and human breast epithelial cell line MCF 10A at varied testing concentrations (**Figure** **4**A). In contrast, the toxicity of Y@LDH was triggered in a TME‐mimicking culture medium (pH = 6.5, [Cys] = 3 mM), and a typical dose‐dependent inhibition to murine mammary carcinoma cells 4T1 was observed (Figure 4B). The results of live/dead staining and colony formation assay were consistent with the cell viability assay, in which Y@LDH caused the highest level of cell death and proliferation inhibition in the TME‐mimicking culture medium (Figures S19 and S20, Supporting Information). It is noteworthy that the concentration of LDH in Y@LDH can only reach a maximum of 5 µg·mL^−1^, and at this concentration, LDH exhibited only marginal inhibition to 4T1 cells, regardless of whether the pH was 7.4 or 6.5, and whether or not Cys was present, in the absence of H2S (Figure S21, Supporting Information). Therefore, the significant difference in toxicity between Y@LDH and yeast was mainly attributed to the TME‐triggered inter‐cooperation between yeast and LDH. To further verify this process, naphthalene‐2,3‐dicarboxaldehyde (NDA) was used to detect intracellular thiols. Both yeast and Y@LDH treatments resulted in a clear decrease in fluorescence intensity, indicating a competitive depletion of intracellular thiols by yeast (Figure 4C). In addition, the quantified concentration of intracellular thiols after Y@LDH treatment was determined to be only one‐fifth of that in the control group (Figure S22, Supporting Information), confirming the thiol competitive depletion by yeast in Y@LDH. Furthermore, FerroOrange was used to trace the changes of intracellular Fe^2+^. As expected, Fe^2+^ content elevated in cells treated with Y@LDH (Figure 4D), indicating the formation and uptake of CoFe(II)S. This phenomenon was also observed by using bio‐TEM. After LDH and Y@LDH treatments, the cargo in endosomes of 4T1 cells showed distinct differences and was consistent with the morphology of LDH and amorphous CoFe(II)S, respectively (Figure 4E). Moreover, due to the lack of catalase in most mitochondria, GSH is regarded as the most dominant reductant in mitochondrial metabolism.^[^ ^31^ ^]^ The competitive depletion of thiols (including GSH and Cys) and uptake of CoFe(II)S with highly catalytic properties cooperatively led to an apparent increase of ROS (Figure S23, Supporting Information), a decrease of mitochondrial membrane potential (Figure S24A, Supporting Information), and severe DNA damage (Figure S24B, Supporting Information). Furthermore, the anti‐migration performance of Y@LDH on 4T1 cells was also proved by a scratching assay according to previous protocols (Figure S25, Supporting Information).^[^ ^32^ ^]^

Antigenicity is another feature of Y@LDH. *β*‐glucan, the main ingredient of yeast cytoderm, is a known immune stimulant.^[^ ^33^ ^]^ The binding of *β*‐glucan with Dectin‐1/TLR‐4 outside macrophage cell membranes promotes M1 polarization and stimulates the release of pro‐inflammatory cytokines.^[^ ^34^ ^]^ To evaluate the influence of Y@LDH on macrophage polarization, nitric oxide, a key feature of M1 polarization,^[^ ^35^ ^]^ was chosen as an indicator. After incubating macrophages with Y@LDH, the NO concentration in the culture medium significantly elevated, reflecting the M1 polarization of macrophages (Figure 4F). The morphological expansion and intracellular NO staining also supported this result (Figure 4G and Figure S26, Supporting Information). Meanwhile, the elevated NO level after LDH treatment could be attributed to the influence of iron ions, which also caused the higher NO concentration of the Y@LDH‐treated group than the yeast‐treated group.^[^ ^36^ ^]^

Taken together, the inter‐cooperative process of Y@LDH biohybrid has been proved in a TME‐mimic environment. Y@LDH effectively inhibited tumor cell proliferation and migration by competitively depleting GSH and in situ production of cytotoxic CoFe(II)S (Figure 4H). Additionally, due to the antigenicity of yeast and the influence of iron ions, Y@LDH could polarize macrophage into M1 type, thereby activating the immune system.

### 2.4 In Vivo Biosafety

Ensuring biosafety is of utmost importance when investigating the therapeutic microorganism. Based on modified Karber analysis, the median lethal dose (LD_50_) of yeast and Y@LDH was calculated to be 5.72 × 10^6^ and 5.04 × 10^6^ CFU g^−1^, respectively (Figure S27A, Supporting Information). The therapeutic dosages of yeast and Y@LDH used in this study were much lower than the calculated LD_50_, indicating that they would not pose a threat to the life of mice. Additionally, the LDH modification had a shielding effect on the yeast surface, which reduced the antigen‐induced rise of white blood cell concentration one day after intravenous injection (Figure S27B, Supporting Information). White blood cell concentration recovered within 7 days and red blood cell concentration maintained within the normal range after the injection of Y@LDH (Figure S27C, Supporting Information).

To assess the liver and kidney function after Y@LDH treatment, we selected alanine aminotransferase (ALT), aspartate aminotransferase (AST), alkaline phosphatase (ALP), blood urea nitrogen (BUN), uric acid (UA), and creatinine (CR) as biomarkers. All of these biomarkers fluctuated within the normal range in 7 days (Figure S27D,E, Supporting Information). Furthermore, the main organs were collected from mice and sliced for histological analysis 30 days after the injection of Y@LDH. Only the lung exhibited a few morphological changes (Figure S28, Supporting Information). These changes, however, were almost inevitable for microorganisms of micron size due to the extremely small size of lung capillaries. These results suggest that the routine therapeutic dosage of Y@LDH will not cause severe side effects and is suitable for further in vivo anticancer experiments. Taken together, our data demonstrate that Y@LDH is a safe and promising therapeutic candidate for cancer treatment.

### 2.5 In Vivo Anti‐Tumor Properties

**Figure 5:**
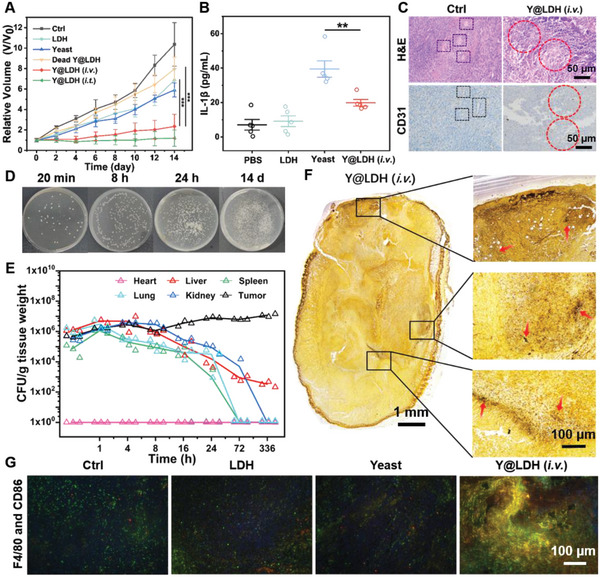
In vivo anti‐tumor properties of Y@LDH. A) Tumor volume of mice from different treatment groups (*n* = 6). B) IL‐1*β* level in sera from mice isolated on day 1 before the second treatment. C) H&E staining and immunohistochemistry staining of CD31 of tumor slices 1 day after injection of Y@LDH. Black rectangle: intact blood vessels, red circle: destroyed blood vessels. D) Dilution coating on NYDB agar plates to evaluate the colonization of Y@LDH in tumors and E) quantification of Y@LDH in various organs harvested from 4T1‐bearing mice at different time points after injection of Y@LDH (partial data points are zeroed and overlapped at the bottom). F) Grocott–Gomori's methenamine silver staining of tumor slice to exhibit the colonization of Y@LDH. Red arrows: yeast. This panoramic image of the tumor is made up of several small images. G) Immunofluorescent staining of F4/80^+^ CD86^+^ M1 macrophages of tumor histologic sections on 14^th^ day (green: F4/80^+^, red: CD86^+^, blue: DAPI). Data are presented as the mean ± SD. Statistical significance was analyzed via the two‐tailed Student's *t*‐test (***p < 0.001, **p < 0.01, and *p < 0.05).

For in vivo antitumor assessment, the subcutaneous mouse 4T1 tumor model was constructed. Tumor‐bearing mice were treated with different therapeutic agents three times on days 1, 2, and 3 (Figure S29, Supporting Information). Both intravenous and intratumoral injections of Y@LDH resulted in significant suppression of tumor growth (**Figure** **5**A and Figures S30–S32), while the therapeutic efficacy of dead Y@LDH was limited due to the lack of inter‐cooperation. Additionally, all treatments did not induce any noticeable changes in the body weight of tumor‐bearing mice (Figure S33, Supporting Information). Furthermore, the shielding effect of LDH on yeast antigen was observed during the anti‐tumor process (Figure 5B). The modification of LDH relieved the infection‐induced elevation of interleukin‐1*β* (IL‐1*β*), which is the fast‐responsive inflammatory cytokines in connection with pathogen invasion. This phenomenon reflected less recognition and clearance of microorganisms by white blood cells, thereby enhancing the delivery efficiency of Y@LDH.

Following Y@LDH treatment, erythrocyte outflow and vascular incompleteness were observed in H&E staining and CD31 immunohistochemistry staining of tumor slices, respectively, along with an elevation of histamine in blood serum (Figure 5C, and Figures S34 and S35, Supporting Information). These results indicated the targeting and infiltration of Y@LDH towards tumor sites and explained the comparable efficacy of intravenous and intratumoral injections. Thereafter, major organs and tumor tissues from mice were collected and weighed at different time points after the Y@LDH injection. All samples were then triturated, diluted, and plated on NYDB plates. Colony‐forming units in plates represented the yeast concentration in corresponding organs. Consistent with the fact that tumor tissues are immunosuppressive, yeast exhibited a sustained increase in population only at the tumor site (Figure 5D,E and Figure S36, Supporting Information). The colonization of Y@LDH was also been observed through Grocott–Gomori's methenamine silver staining. After methenamine silver staining, fungi were stained black. Several yeast enrichment regions appeared in the tumor slice on day 14 after the injection of Y@LDH (Figure 5F), while few fungi could be seen in other organs (Figures S37 and S38, Supporting Information).

The effect of Y@LDH platform on local immune activation was further investigated. In our study, the colonization of yeast in tumor tissues could be regarded as an acute introduction of antigens, which were supposed to stimulate resident macrophages to secrete pro‐inflammation cytokines, thereby reactivating the immunosuppressive tumor. Immunofluorescence staining was used to mark the infiltration of various immune cells. Similar to the in vitro results, Y@LDH treatment induced significant promotion of M1 macrophage (F4/80^+^ CD86^+^) infiltration and reduction of M2 macrophage (F4/80^+^ CD163^+^) content in tumor tissues (Figure 5G and Figure S39, Supporting Information). Sole LDH or yeast injection, however, did not present a similar phenomenon as they did in macrophage polarization experiments and exhibited almost no difference from PBS injection. This is potentially attributed to the absent acid‐targeting ability of LDH and the absent antigen shield of yeast. Meanwhile, activation of the immune system was also reflected by the remarkable promotion of T effector cells (CD8^+^) and T helper cells (CD4^+^) after Y@LDH treatment in tumor tissues (Figures S40 and S41, Supporting Information), as well as the enhanced proportion of mature DC cells (CD11c^+^ MHCII^+^ CD80^+^/CD86^+^) in tumor‐draining lymph nodes (Figure S42, Supporting Information). The reactivation of immunosuppression was believed to be an important factor for the anti‐tumor properties of Y@LDH.

### 2.6 Immune Activation

**Figure 6:**
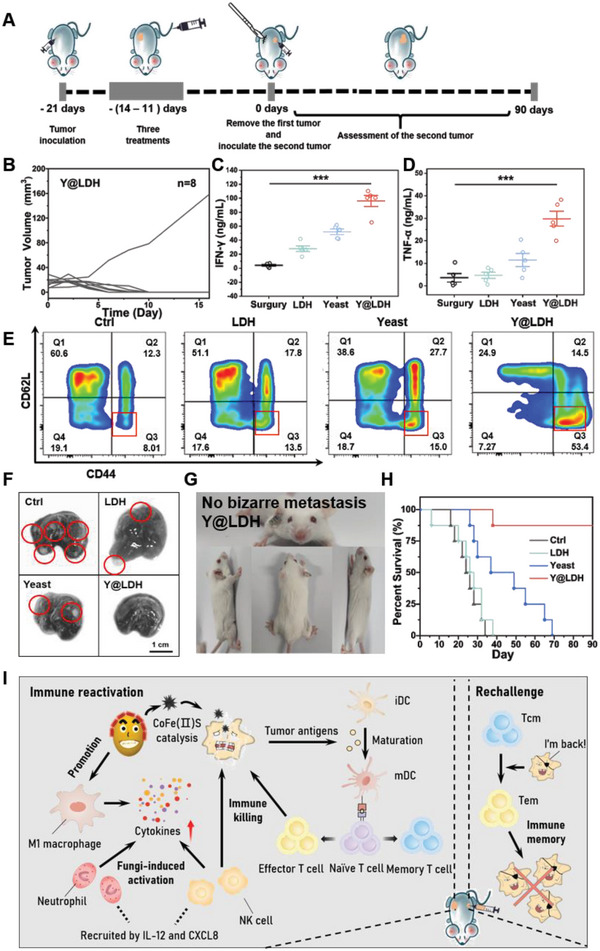
In vivo immune response properties of Y@LDH. A) Schematic illustration to evaluate the immune response of mice after different treatments. B) Second tumor growth curves of each mouse in the Y@LDH group. C) IFN‐*γ* and D) TNF‐*α* levels in sera from mice isolated 1 day after the second tumor rechallenge. E) Representative flow cytometry plots of CD8^+^ effector memory T cells in the spleen induced by different treatments on mice bearing 4T1 tumors after the second tumor rechallenge (Gate1:CD45, Gate2:CD8). F) Representative photographs of the ink‐stained lungs from mice after different treatments. G) Representative photographs of mice post Y@LDH treatment. H) The survival curves of mice up to 90 days after various treatments indicated (*n* = 8). I) Schematic illustration of immune response triggered by Y@LDH. Data are presented as the mean ± SD. Statistical significance was analyzed via the two‐tailed Student's *t*‐test (***p < 0.001, **p < 0.01, and *p < 0.05).

The efficacy of immune reactivation by using certain microorganisms has been investigated to match that of immune checkpoint therapy.^[^ ^37^ ^]^ To further explore the immune memory effect induced by Y@LDH, a re‐challenged tumor model was used (**Figure** **6**A). It is interesting to observe that the second tumors on most of the mice after Y@LDH treatment did not exhibit remarkable growth, and in fact were eliminated in 10 days (Figure 6B). After 30 days, the sizes of the spleen and draining lymph nodes in the Y@LDH group recovered their normal size (Figure S42, Supporting Information), while those in other groups were hypertrophy, which could be attributed to the tumor‐caused chronic inflammation. Elevated levels of IFN‐*γ* and TNF‐*α* in serum 1 day after the re‐challenge verified that such an intensive growth inhibition came from the immune response of mice (Figure 6C,D). Furthermore, since the invasion of yeast could also lead to the formation of central memory T cells (T_CM_) with yeast antigen recognition function, analyzing the proportion of T_CM_ after removing the first tumors was deemed less meaningful. In order to confirm the formation of tumor cell‐recognizing memory T cells, we collected spleens from each group for flow cytometry analysis one day after the re‐challenge. The percentage of effector memory CD8^+^ T cells (T_EM_, CD45^+^, CD8^+^, CD62L^−^, CD44^+^) over all CD8^+^ T cells remarkably increased (Figure 6E), which confirmed the immune memory effect against tumor cells. Meanwhile, CD8^+^ T cells (CD45^+^ CD8^+^) and effector memory CD8^+^ T cells (CD45^+^, CD8^+^, CD62L^−^, CD44^+^) are highly expressive in the draining lymph nodes of the second tumors after Y@LDH treatment (Figure S43, Supporting Information). It is also noteworthy that an excellent anti‐metastasis property of Y@LDH was detected. Due to the lengthy experimental period, apart from the normal lung metastasis (Figure 6F and Figure S44, Supporting Information), some bizarre metastasis sites were also found in tumor‐bearing mice except those received Y@LDH treatment (Figure 6G and Figure S45, Supporting Information). Owing to the effective immune memory effect, most mice in the Y@LDH group survived for over 90 days post the re‐challenge and still seemed healthy till this paper was finished, while mice in other groups gradually died within 70 days (Figure 6H). Another meaningful phenomenon is that although the injection of native yeast could not cure the first tumors, it obviously prolonged the survival time of mice after the re‐challenge. This process is alike the effect of bacillus Calmette–Guerin in prostate cancer treatment and is worth further research, from the author's perspective.

## 3 Conclusion

In summary, we designed and successfully constructed Yeast@CoFe LDH‐S_2_O_3_^2−^ as an inter‐cooperative therapeutic platform based on the sulfur metabolism of yeast. After intravenous injections, yeast carried LDH into tumor tissues by the acid‐targeting ability. TME then triggered the release of S_2_O_3_^2−^ by LDH and the production of H_2_S by yeast. The S_2_O_3_^2−^ released as well as overexpressed thiols in TME served as sulfur resources for yeast to produce H_2_S. And H_2_S reacted with LDH in return, producing CoFe(II)S. The reactions also promoted the liberation of LDH and the further release of S_2_O_3_^2−^. The product, CoFe(II)S, was proved to present strong catalytic properties and could effectively catalyze H_2_O_2_ to hydroxyl radicals with high cytotoxicity. GSH competitive depletion by yeast further enhanced the cytotoxicity of CoFe(II)S. Meanwhile, Y@LDH also induced immune reactivation in tumor tissues and intensified the immune memory effect toward tumor cells (Figure 6I).

Although LDH and Baker's yeast seem to be two irrelevant substances in nature, after integration, the two components share the merits of each other and enable agitated tumor inhibition. With the inter‐cooperative design, the combination of living microorganisms and functional materials could jointly enable unique functionalities, which provide applicable methods for microbes and materials whose original efficacy is not suitable to be therapeutics alone. The diverse types of microorganisms, as well as functional nanosubstances, may provide abundant choices for the design of inter‐cooperative platforms, enabling many unique advantages and strong therapeutic efficacy which are hard to achieve. This study is therefore anticipated to spark another wave in the follow‐on exploration of multifunctional therapeutics based on this concept.

## Conflict of Interest

X.L. and F.L. are inventors on several pending patents related to the device and design described here, filed by the China National Intellectual Property Administration (2022100146563; 2022100146563). The authors declare that they have no other competing interests.

## Supporting information

Supporting Information

## Data Availability

The data that support the findings of this study are available on request from the corresponding author. The data are not publicly available due to privacy or ethical restrictions.
