# Supplementary material for: An Inter‐Cooperative Biohybrid Platform to Enable Tumor Ablation and Immune Activation
Source: Adv Sci (Weinh). 2023 Jun 14;10(23):2207194. doi: 10.1002/advs.202207194 (PMC10427385; doi:10.1002/advs.202207194)
Supplement: Supplementary file 1 — Supporting Information [file ADVS-10-2207194-s001.pdf]

ADVANCED SCIENCE
Open Access

Supporting Information

for *Adv. Sci.*, DOI 10.1002/advs.202207194

An Inter-Cooperative Biohybrid Platform to Enable Tumor Ablation and Immune Activation

Feiyu Li, Qiang Chu, Zefeng Hu, Zijie Lu, Chao Fang, Gaorong Han, Yike Fu and Xiang Li**

Supporting Information

An Inter-cooperative Biohybrid Platform to Enable Tumor Ablation and Immune Activation

Feiyu Li, Qiang Chu, Zefeng Hu, Zijie Lu, Chao Fang, Gaorong Han, Yike Fu *, *Xiang Li**

F. Li, Z. Hu, Y. Fu, X. Li

Zhejiang Univ, Sch Mat Sci & Eng, State Key Lab Silicon Mat, Hangzhou 310027, People's Republic of China.

ZJU-Hangzhou Global Sci and Technol Innovat Center, Zhejiang University, Hangzhou 311215, People's Republic of China.

E-mail: fyk3927@zju.edu.cn (Y. F.); xiang.li@zju.edu.cn (X. L.)

Z. Lu, C. Fang, G. Han

Zhejiang Univ, Sch Mat Sci & Eng, State Key Lab Silicon Mat, Hangzhou 310027, People's Republic of China.

Q. Chu

Zhejiang Univ, Coll Agr & Biotechnol, Tea Res Inst, Hangzhou 310058, People's Republic of China.

Experimental Procedure

Materials

Iron nitrate nonahydrate ($Fe(NO_3)_3 \cdot 9H_2O$, 99.0%), sodium hydroxide (NaOH, 98.0%), sodium thiosulfate pentahydrate ($Na_2S_2O_3 \cdot 5H_2O$), ferric chloride hexahydrate($FeCl_3 \cdot 6H_2O$, 99.0%), hydrogen peroxide (H_2O_2, 30%) and glucose were obtained from Sinopharm Chemical Reagent Co., Ltd. Cobalt nitrate hexahydrate ($Co(NO_3)_2 \cdot 6H_2O$, >99%), 3, 3′, 5, 5′- tetramethylbenzidine (TMB, >99.0%), N,N-dimethyl-p-phenylenediamine dihydrochloride, DMPO and DCFH-DA (≥97%) were purchased from Sigma Aldrich. Agar and yeast extracts were obtained from Shanghai Yuanye Bio-Technology Co., Ltd. Beef extract were purchased from Aladdin Reagents Co., Ltd. Transwell@ insert were purchased form Corning Inc. 5,5'-Dithiobis-(2-nitrobenzoic acid) (DTNB) was acquired from Meryer. Hochest 33258, calcein AM, propidium iodide, Rhodamine123, NO probe, NO testing kits, ethylene diamine tetraacetic acid (EDTA), radio immunoprecipitation assay (RIPA) lysis buffer, phenylmethylsulfonyl fluoride (PMSF) were obtained from Beyotime Biotechnology. FerroOrange

was obtained from Dojindo. Naphthalene-2,3-dicarboxaldehyde (NDA) was bought from Invitrogen™. All chemicals in this work were used directly without further purification.

Characterization

The morphology of samples was observed by a field-emission scanning electron microscope (FESEM; SU-70 Hitachi) and a transmission electron microscope (TEM, FEI Tecnai F20). High angle annular dark field images and element mapping were obtained on a Cs-corrected STEM (FEI Titan G2 80-200 C hemi STEM). The crystal structure was characterized by X-ray diffraction with Cu Kα radiation (XRD, X'pert PRO MPD). The size distributions and zeta potentials were tested by a Malvern zetasizer (Nano-ZS90, UK). All the UV-vis spectra were measured by a UV-vis spectrometer (Shimadzu, UV-2600). The fluorescence images were photographed by an inverted fluorescent microscope (Nikon, Ts2R-FL).

Synthesis of $S_2O_3^{2-}$ intercalated CoFe layered double hydroxides

Briefly, 0.6 mmol $Co(NO_3)_2 \cdot 6H_2O$ and 0.2 mmol $Fe(NO_3)_3 \cdot 9H_2O$ were dissolved in 20 mL of deionized water as solution A. Meanwhile, 8 mmol NaOH was dissolved in another 20 mL of deionized water to make solution B. Afterwards, under 520-rpm stirring at 65°C, solution A and B were dropwise added simultaneously into a beaker containing 0.1 mmol $Na_2S_2O_3 \cdot 5H_2O$ and 80 mL of deionized water. pH was kept around 9.5. Afterwards, the solid red-brown precipitants were collected by centrifugation, and then washed twice with the mixture of deionized water and ethanol (volume ratio: 5:1). It should be noted that all the deionized water should be boiled before use to remove dissolved carbon dioxide and the synthesis process should be accomplished within 20 min to prevent the formation of Co/Fe oxides.

Yeast culture medium preparation and yeast culture

10 g of glucose, 5 g of yeast extract and 8 g of beef extract were mixed in 1 L of deionized water to prepare liquid NYDB culture medium. For solid NYDB medium, additional 20 g of agar was added. Baker's yeast was purchased in Yonghui supermarket in China and the brand is 'Angel'. In order to purify baker's yeast, the commercial yeast was diluted in PBS, streaked on the solid medium plate and then incubated at 37 °C overnight. Yeast colonies were then picked out and grown overnight in NYDB liquid medium in a shaking incubator (37°C, 180 rpm) before use.

Characteristics of Y@LDH

To load LDH on Yeast, 10^7 CFU yeast and different quantities of LDH were dispersed in 10 mL of deionized water. In this work, the quantity of LDH in mixture was finally decided as 0.5 mg. After stirring for 2 h, Y@LDH was collected by centrifugation and then re-suspended in ultrapure water for further use.

Culture experiment and colony model

Same yeast concentration of yeast and Y@LDH were both incubated in pH=7.4 and pH=6.5 RPMI-1640 culture medium (with 10% fetal bovine serum, pH was adjusted by HCl), respectively. Due to the influence of LDH on OD600, which was used to judge the concentration of yeast, we use Δ concentration (from ΔOD600) to estimate the influence of LDH on yeast proliferation. For colony model, same yeast concentration of yeast and Y@LDH were directly coated on NYDB solid plates and the photograph were taken after colony formation.

Catalytic Property

In this work, 3, 3′, 5, 5′-tetramethylbenzidine (TMB, 1×10^{-3} M) was chosen as an indicator to detect the production of reactive oxygen species (ROS), following the relative procedures reported previously.[1] TMB can be oxidized to ox-TMB (λ=652 nm) by hydroxyl radicals (·OH). To separate the influence of some enzymes like POD outside yeast cell membranes on catalytic property, all samples were boiled at 70 °C for 10 min to inactivate the catalytic properties of yeast enzymes. DMPO was used to capture radicals. The combination of DMPO and hydroxyl radicals shows a 1:2:2:1 signal on ESR spectroscopy. To further clarify the enzyme-like activity of Y@LDH, Michaelis-Menten kinetic curve was drawn by plotting the initial ·OH producing velocities on different H_2O_2 concentrations, and the Michaelis-Menten constant (K_M) and maximal velocity V_{max} were then calculated. It should be noted that before the final test for LDH with different concentrations of Na_2S, the product should be centrifuged and washed for two times.

H_2S production

Different concentrations of yeast or Y@LDH with Cys/GSH were added into 20 mL glass bottle with 10 mL of antibiotic-free RPMI 1640 medium to simulate body fluid or tumor tissues with additional acid. A rubber tube was used to link this bottle with another bottle containing 2 mL of zinc acetate/sodium acetate mixture (mass ratio: 4/1) as testing bottle. All joints were sealed by Vaseline and laboratory film. The whole device (as shown in Figure S10) was then kept in a shaking incubator

(37°C, 180 rpm). After specific time, N, N-dimethyl-p-phenylenediamine dihydrochloride and $FeCl_3$ were added into the testing bottle. After incubation for another 15 min, the absorbance at 665 nm (corresponding to the production of methylene blue) of the mixture was examined, and the concentration of H_2S was determined using a standard curve of Na_2S.

$S_2O_3^{2-}$ release

A typical chemical titration process was used for detecting $S_2O_3^{2-}$ released. Briefly, 2.5 mg mL^{-1} CoFe LDH-S_2O_3 was dispersed in pure PBS and PBS added with acid or Na_2S, respectively. At specific time points, materials were centrifuged and the supernatant containing $S_2O_3^{2-}$ was used for titration based on following reaction:

$$C_2O_7^{2-} + 6I^- + 16H^+ \rightleftarrows 2Cr^{3+} + 7H_2O + 3I_2$$

$$I_2 + 2S_2O_3^{2-} \rightleftarrows 2I^- + S_4O_6^{2-}$$

Specifically, 60 mg of potassium dichromate was dissolved by 20 mL of ultrapure water in an iodine measuring bottle. Then 0.7 g of potassium iodide and 20 mL of 20% sulfuric acid were successively added and gently shaken to dissolve. After being sealed in the dark for 10 min, 150 mL of water was added and then 10 mL of diluted mixture was taken out as the indicator for titration. The supernatant was titrated with a burette. When the titration was near the end point, 2 mL of 10 mg mL^{-1} starch indicator solution was added. Titration process continued till the blue color disappeared and the volume of final titration solution was recorded. The colors changed during the titration can be showed in UV-vis spectra as Figure S8. The concentration of $S_2O_3^{2-}$ in supernatant can be calculated as follows:

$$c(S_2O_3^{2-}) = \frac{m \times 1000}{\Delta V \times M}$$

Where m is the mass of $K_2Cr_2O_7$ in indicating solution (g), ΔV is the volume of supernatant used during titration (mL), and M is the molar mass of $K_2Cr_2O_7$ (M=294.18 g mol^{-1}).

Chemotaxis characteristic of yeast and Y@ LDH

The chemotaxis of yeast and Y@LDH was investigated via a Transwell[@] migration assay (8 μm pore size, 6.5 mm diameter). To simulate the acidic tumor microenvironment, 800 μL RPMI 1640 medium with 10% fetal bovine serum (FBS) was adjusted to pH 6.0 and added to the bottom of 24-well plate.

For the control group, 800 μL RPMI 1640 medium with 10% FBS was directly added to the bottom. Thereafter, yeast or Y@LDH (200 μL, 5×10^5 CFU mL^{-1}) were added to the upper Transwell insert. At 10, 20 and 30 min, the Transwell[@] inserts in specific group were removed and then let wells stand for 10 min. Afterwards, the bottom of each well was photographed and the quantities of yeast were counted.

In vitro study

Murine breast cancer (4T1) cells, murine liver (AML12) cells, human breast epithelial (MCF 10A) cells and mononuclear macrophage (RAW 264.7) cells were cultured with RPMI 1640 or DMEM medium containing 10% FBS at 37 °C in 5% CO_2 atmosphere for *in vitro* assays.

Cytotoxicity assay

For each treatment, 4T1 cells were digested and seeded into a 24-well plate at the density of 15000 cells per well and incubated for 12 h. Except cell safety assay, all cytotoxicity experiments were carried in hypoxia condition (5% O_2) to simulate the hypoxia environment in tumor tissue. Subsequently, Transwell[@] inserts (0.4 μm pore size, 6.5 mm diameter) were laid and fresh culture medium containing different therapeutic agents (LDH, Yeast and Y@LDH) was added to the inserts. The pH and Cys concentration of medium were adjusted to meet different experimental requirements. After incubating for another 24 h, 500 μL of 10% CCK-8 was added to each well and the absorbance at 450 nm was detected by a microplate reader. The viability of each group was calculated based on the corresponding absorbance over those of control groups.

Colony formation assay

4T1 cells were seeded into a 24-well plate at the density of 50 cells per well. After 4 h for cells adherence, PBS, LDH, Yeast and Y@LDH were added and incubated for another 12 h, respectively. Then, the cells were cultured in fresh RPMI 1640 medium for 14 d. Afterwards, the cells were fixed by 10% formaldehyde (10 min) and stained by crystal violet (20 min).

Scratching assay

Sufficient 4T1 cells were seeded into 24-well plates and incubated for 24 h. Sterile toothpick was used to scratch a line on the bottom of each plate. Subsequently, PBS was used to wash away dead cells for three times. Thereafter, PBS, LDH, Yeast and Y@LDH were added to 400 nm pore-size

Transwell[@] inserts on each well and incubated for 12 h, respectively. Cells were photographed by microscope every 12 h.

Fluorescence staining

In this part, different fluorochromes were used to characterize the influence of therapeutic agents on cells, respectively. Briefly, after different treatments, cells were washed with PBS for three times. Then, Hoechst 33258 (3 μg mL^{-1}), NDA (10 μM), FerroOrange (5 μM), DCFH-DA (2.5 μM), Rhodamine123 (2 μM), calcein AM (4 μM) and propidium iodide (PI) (8 μM) were added to detect the corresponding indexes, respectively. After incubation for further 30 min, the cells were washed and observed with inverted fluorescence microscope. In some groups, DAPI was used to localize the cell nuclear.

Intracellular GSH quantification

Sufficient 4T1 cells were seeded in 6-cm culture dishes and incubated for 24 h. Then PBS and Y@LDH were added, respectively. After incubation for further 8 h, the cells were washed for three times and 500 μL of RIPA lysis buffer with PMSF (10 mM) was added. To detect the GSH concentration in supernatant, 50 μL of 500 μM DTNB was mixed with 50 μL of supernatant for 5 min and the absorbance at 405 nm was detected by a microplate reader. The concentration was calculated according to the standard curve.

Macrophage polarization

RAW 264.7 was used to indicate the promotion of macrophage polarization by different therapeutic agents (LDH, Yeast and Y@LDH). Nitric oxide probe and nitric oxide kits were chosen to indicate the M1 polarization. Specifically, RAW 264.7 was seeded into 24-well plate of 5000 cells per well and incubated for 12 h. Then Transwell[@] inserts were laid and therapeutic agents were added. After incubation for another 12 h, the supernatant of each group was collected and tested by nitric oxide kits. 20 μM NO probe was then directly added to the remnants and incubated for 30 min. Afterwards, the cells were washed and observed under inverted fluorescent microscope.

In vivo study

Female BALB/c mice (4-6 weeks old) were purchased from Shanghai Laboratory Animal Center. All mice experiments were performed in accordance with the Guidelines for Care and Use of Laboratory Animals of Zhejiang University and approved by the Animal Ethics Committee of Zhejiang

University. The mice were randomly divided and a 12 h light/12 h dark cycle controlled environment was set. The ethically endpoint of tumor volume was set to be 1500 cm^3. Tumor volume was calculated as following formula: volume = (tumor length) × (tumor width)^2 × 0.52. The relative volume of tumor was calculated as the tumor volume in specific day over the tumor volume in day 0.

Median lethal dose investigation

To investigate the median lethal dose of Yeast and Y@LDH for BALB/C mice, different doses of yeast and Y@LDH (4.0, 4.6, 5.2, 5.8, 6.4 $\times 10^6$ CFU g^{-1}) were injected intravenously to mice. Each group consisted of six mice that were injected with same doses of yeast or Y@LDH. The death rate of each group was recorded and the median lethal dose of each group was calculated using the modified Karber analysis, the main formula of which is

$$\log_2 LD_{50} = [\mathrm{Xn} - \mathrm{i} * (\sum \mathrm{m} - 0.5)]$$

Where Xn is the logarithm of dose in the highest corresponding group; i is the difference of logarithmic doses between the two adjacent groups (high dose minus low dose); ∑m is the sum of death rate of each group.

In vivo biosafety evaluation

To evaluate the biocompatibility of therapeutic agents, after injection of LDH, Yeast, Y@LDH (10^7 CFU), the blood samples of healthy mice were collected for blood biochemistry and blood routine analysis at different time points (0, 1, 7, and 14 days, respectively). Major organs (heart, liver, spleen, lung, and kidney) of mice were collected and used for histological analysis at day 30.

Tumor ablation and immune reactivation analysis

After acclimation for 1 week, 0.1 mL (≈10^6) of 4T1 cells were injected into the right side back of each mouse to acquire tumor-bearing mice. When the tumor reached about 100 mm^3, all mice were divided into six groups (6 mice for each group) and applied with various treatments three times in the first, second and third day: 1) PBS, 2) LDH (0.25 mg), 3) Yeast (5×10^6 CFU), 4) Dead Y@LDH (5×10^6 CFU), 5) Y@LDH (5×10^6 CFU, the corresponding weight of CoFe LDH-S_2O_3: 0.25 mg), 6) Y@LDH (*i.t.*, 5×10^6 CFU). Unless noted, all therapeutic agents were injected intravenously. Tumor sizes and mouse weights were measured every 2 days in initial 14 days. On the 15th day, all the mice underwent surgery to remove the first tumor unless it had already been cured. The removed tumors were sectioned and analyzed. For the re-challenge model, mice were then injected with same (≈10^6)

4T1 cells on the left side back. The volumes of the second tumor were then recorded and the immune memory response as well as the anti-metastasis properties were analyzed in the next 90 days. To evaluate the percentage of effector memory T cells, the draining lymph node of the second tumor in different groups were harvested and stained with different antibody. The samples were then analyzed by flow cytometry and the percentages of effector T cells ($CD45^+$ $CD8^+$), conventional T cells ($CD45^+$ $CD4^+$) and T_{EM} ($CD45^+$, $CD8^+$, $CD62L^-$, $CD44^+$) were acquired.

Cytokine detection

His (Elabscience$^@$), IL-1β (Elabscience$^@$), IFN-γ (Elabscience$^@$), TNF-α (Elabscience$^@$) in mouse serum samples were analyzed with ELISA kits according to the vendors' protocols.

Y@LDH colonization experiment

Heart, liver, spleen, lung, kidney and tumor samples from mice were harvested and weighed after injection of Y@LDH at specific time points. All samples were then homogenized at 4°C in sterile PBS (pH = 7.2) by a grinding mill. Then the extracts of these samples were diluted with PBS for specific folds and plated on NYDB solid plates. After 24 h incubation, yeast colonies were counted. The yeast concentration (CFU per gram of tissue) was calculated from colony counts, dilution ration and tissue weights.

Statistical analysis

All results are expressed as means ± SD as indicated. Two-tailed Student's t test was used when more than two groups were compared.

Supporting Figures

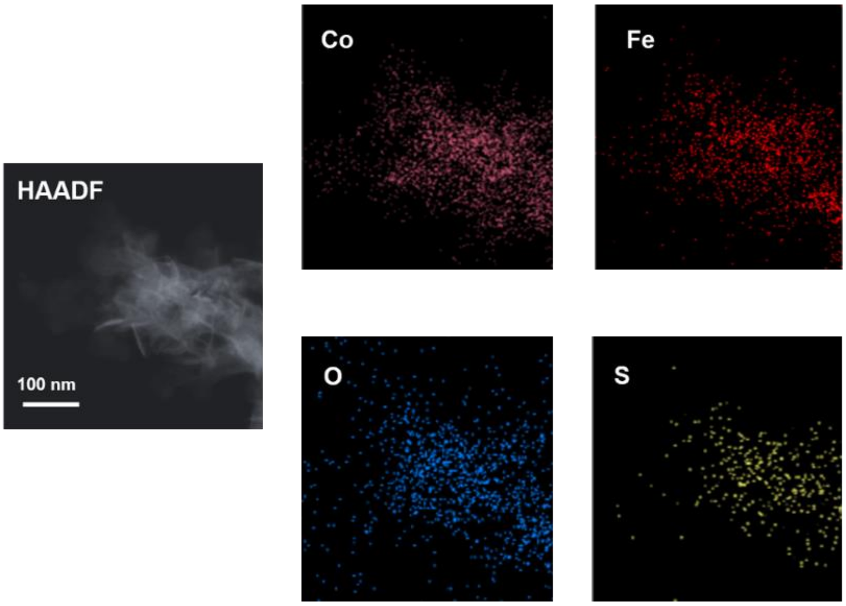

Figure S1. EDS element mapping of LDH.

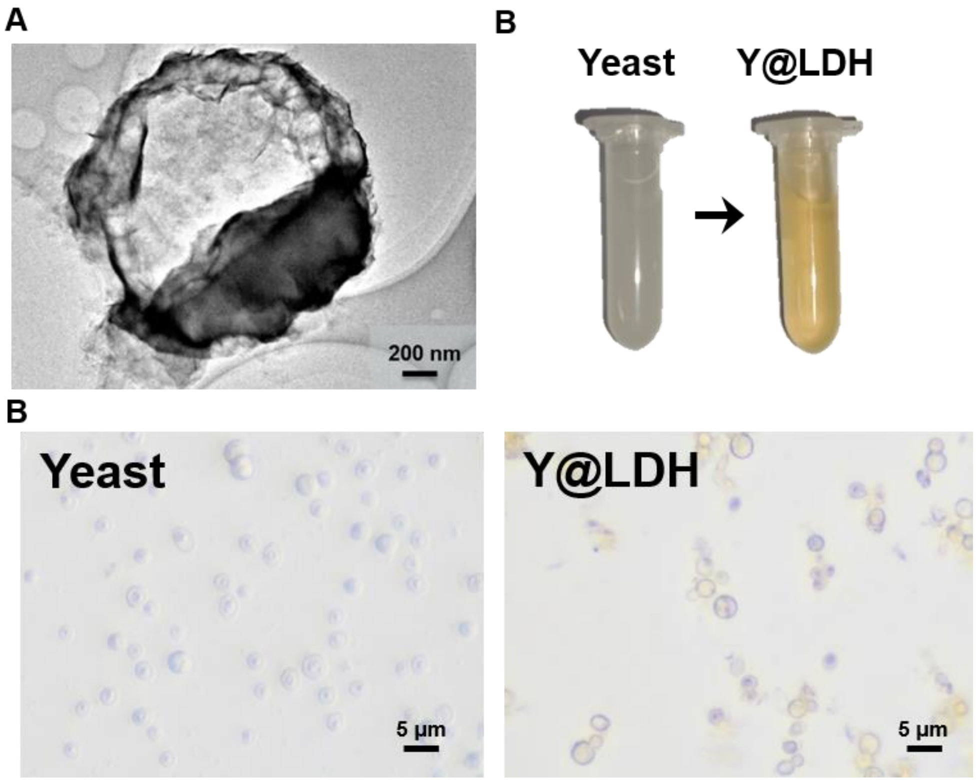

Figure S2. Morphology of Y@LDH and its components. (A) TEM images of Yeast modified by LDH. (B) Optical images of the solutions containing yeast and Y@LDH. (C) Light microscope images of the yeast and Y@LDH.

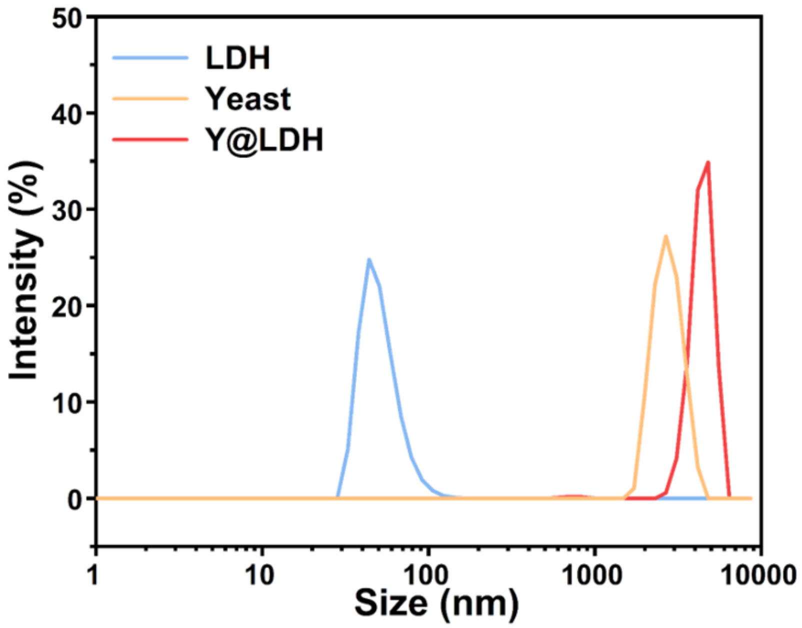

Figure S3. Size distributions of LDH, yeast and Y@LDH.

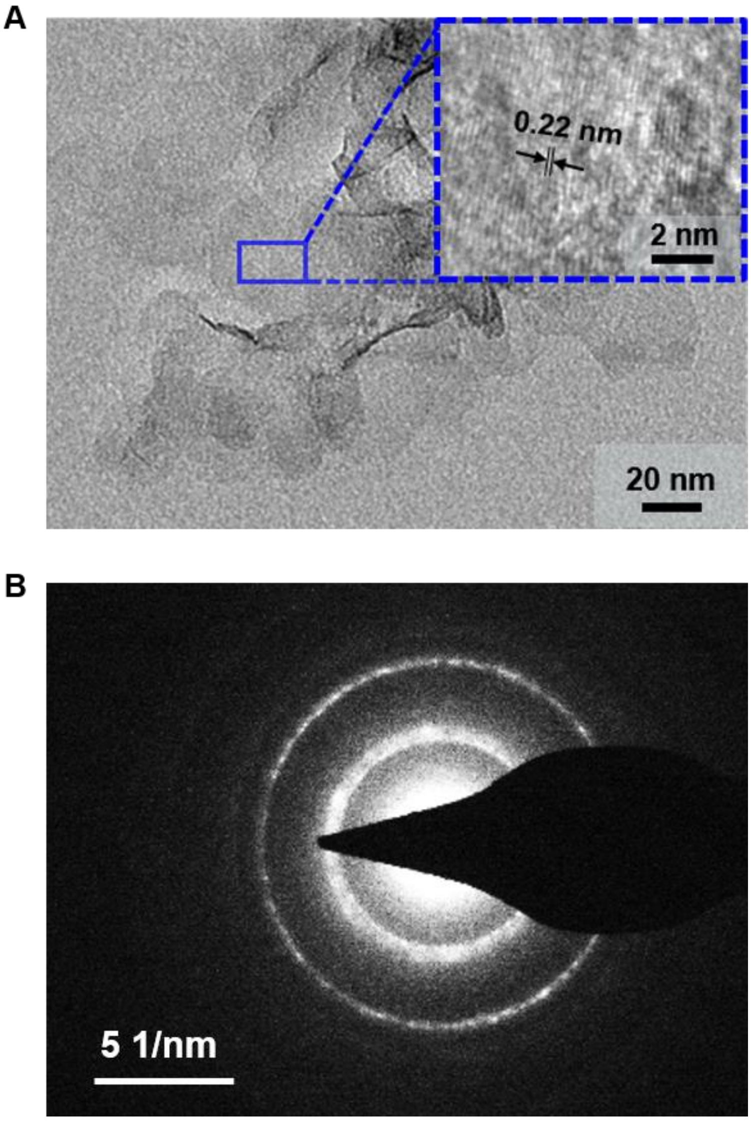

Figure S4. (A) TEM and (B) SAED image of LDH.

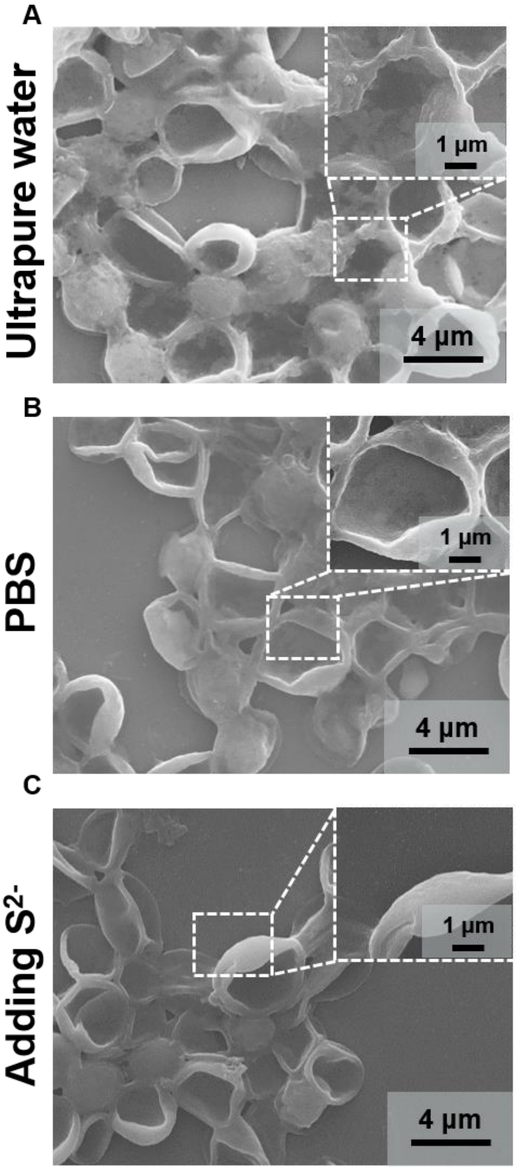

Figure S5. SEM images of Y@LDH in A) Ultrapure water, B) PBS and C) H_2S solution for 6 h.

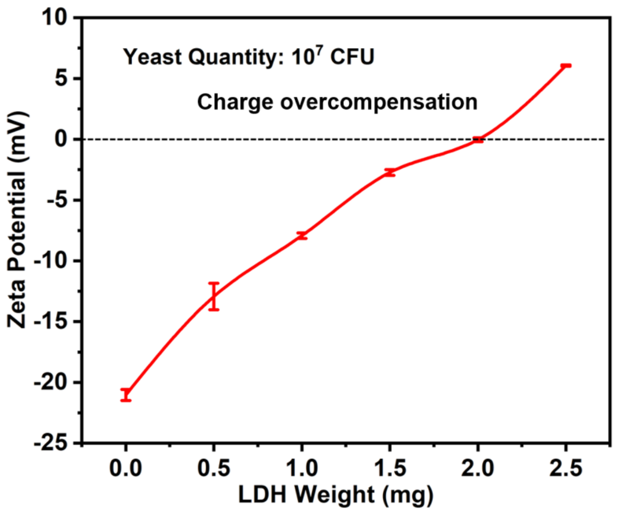

Figure S6. Zeta potentials of yeast loaded with different weight of CoFe LDH-$S_2O_3^{2-}$

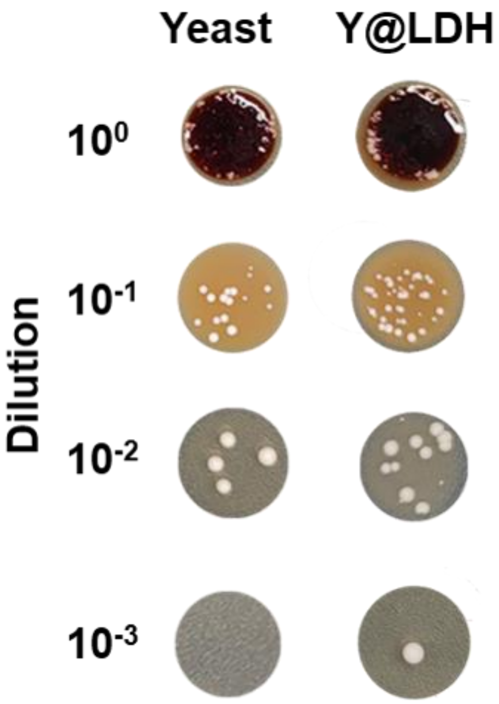

Figure S7. Growth of same amount of Yeast and Y@LDH after mixing with fresh mouse whole blood and plating on NYDB agar plates at different dilutions.

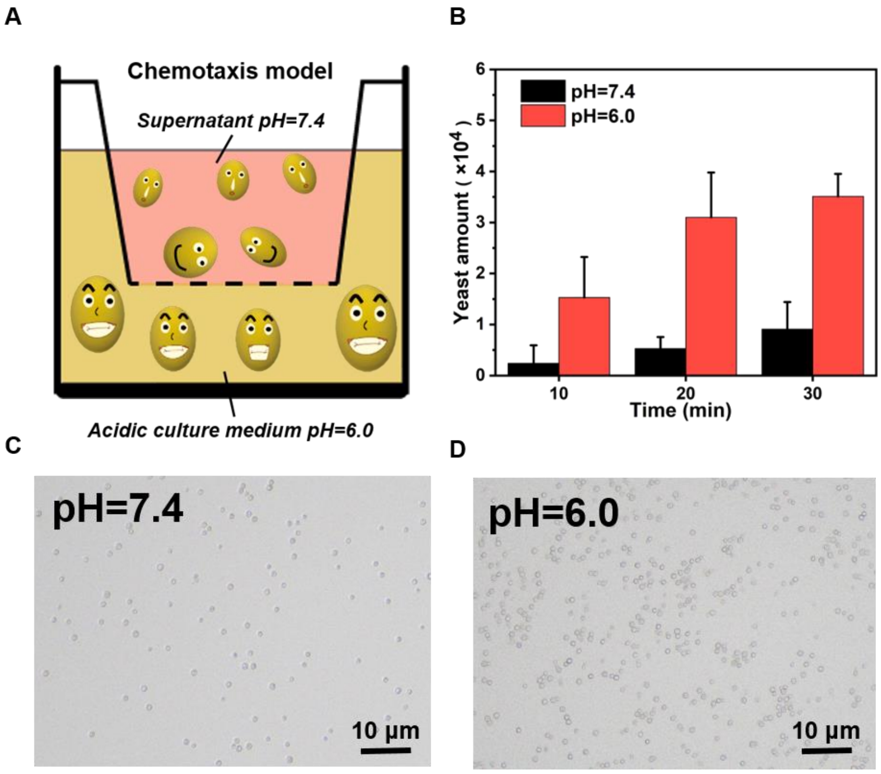

Figure S8. Acid-induced chemotaxis of yeast. A) Schematic illustration of acid-induced chemotaxis model of yeast B) The migration of yeast to the bottom chamber in acid-induced environment. (**C** and **D**) Photographs of chamber bottoms after yeast migration for 30 min in pH=7.4 and 6.0.

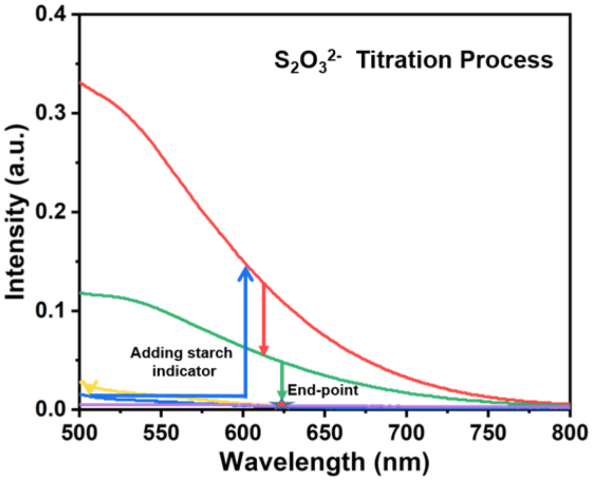

Figure S9. **$S_2O_3^{2-}$ titration process in UV-vis spectra.**

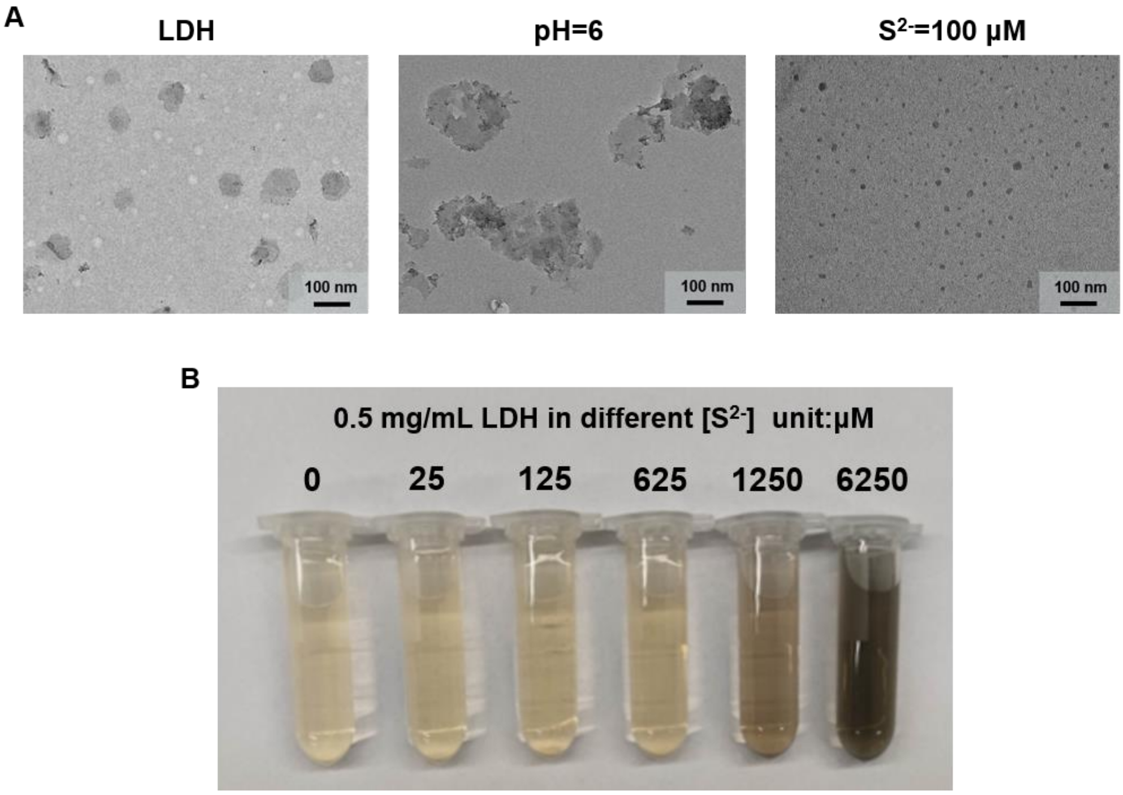

Figure S10. Structural destruction of LDH induced by acid and S^{2-}. A) Morphology change of LDH in acid environment and in the addition of S^{2-} B) Photograph of LDH solution added with different concentration of S^{2-}.

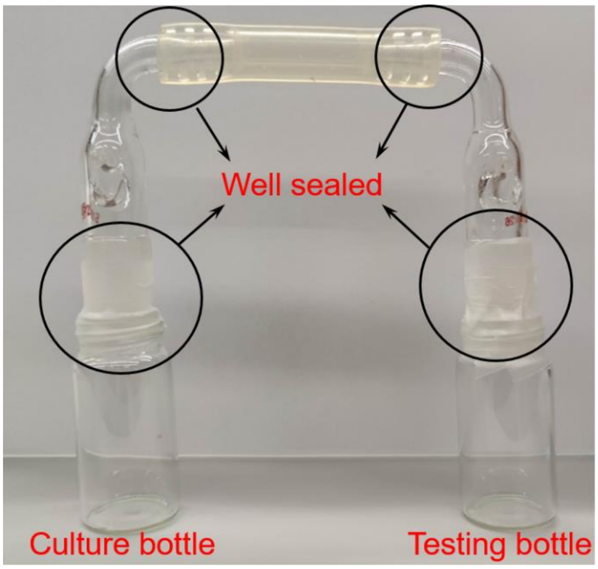

Figure S11. Photograph of the device for detecting the H_2S produced by yeast.

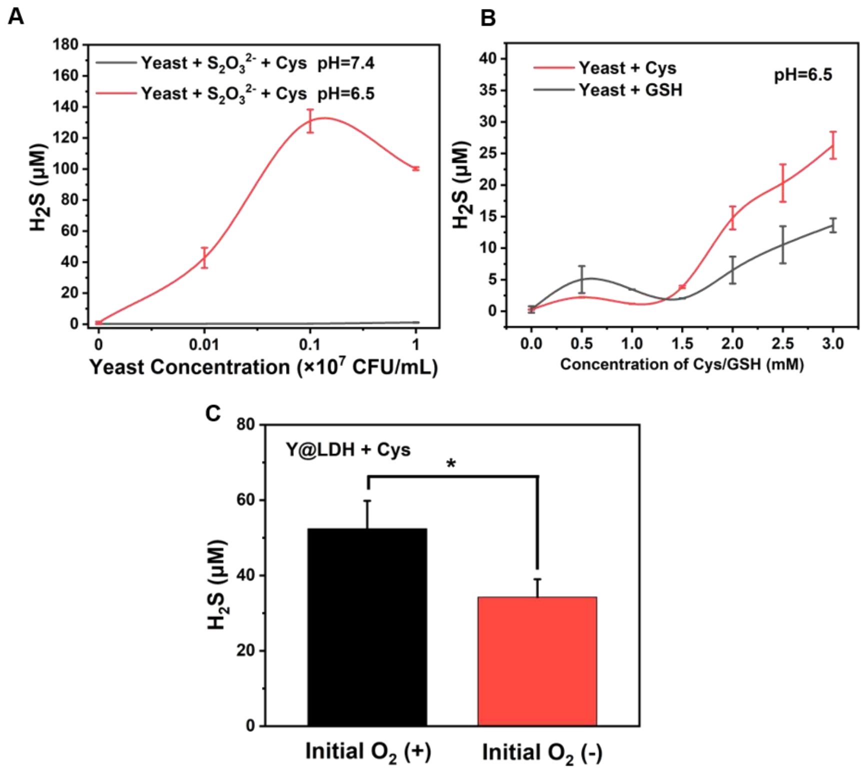

Figure S12. H_2S producing profiles of yeast in different conditions. A) H_2S production of yeast with different concentrations in the solution containing 2 mM $S_2O_3^{2-}$ and 3 mM Cys at pH 7.4 and pH 6.5. B) H_2S production of yeast in the presence of Cys/GSH with different concentrations. C) H_2S production of Y@LDH + Cys with and without initial oxygen supply.

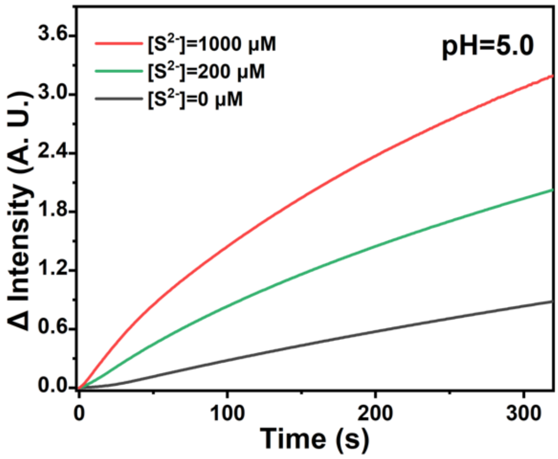

Figure S13. The catalytic properties of LDH with different concentrations of Na2S at pH= 5.0.

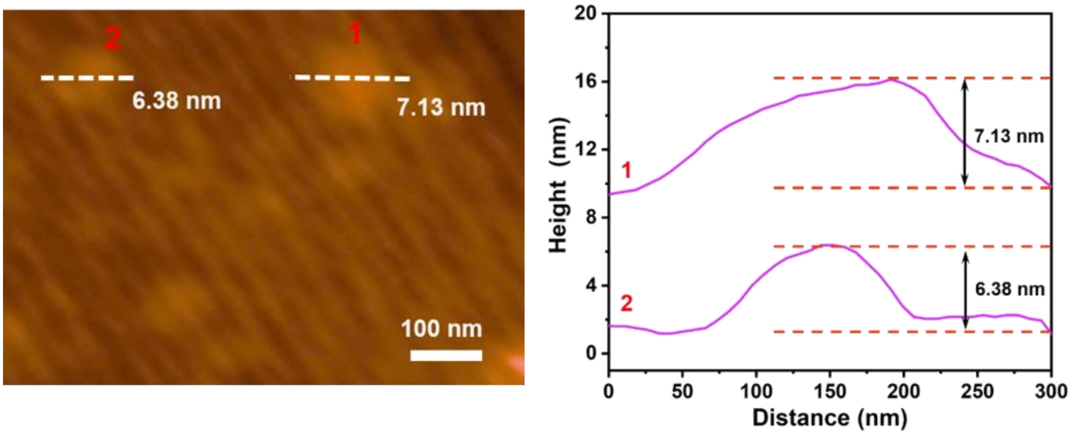

Figure S14. AFM image and height profiles of LDH.

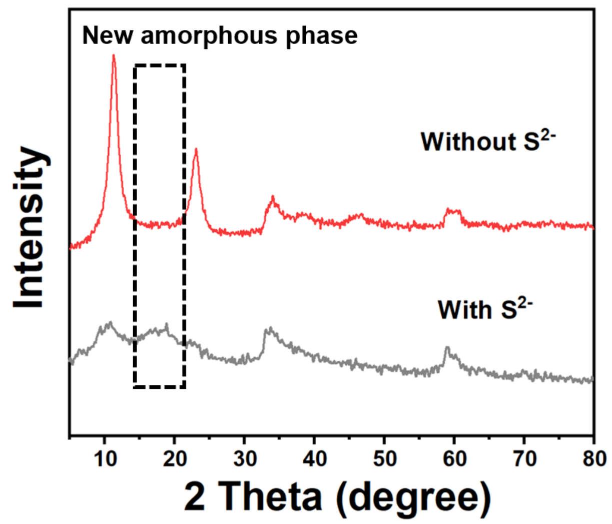

Figure S15. XRD patterns of LDH before and after adding Na_2S.

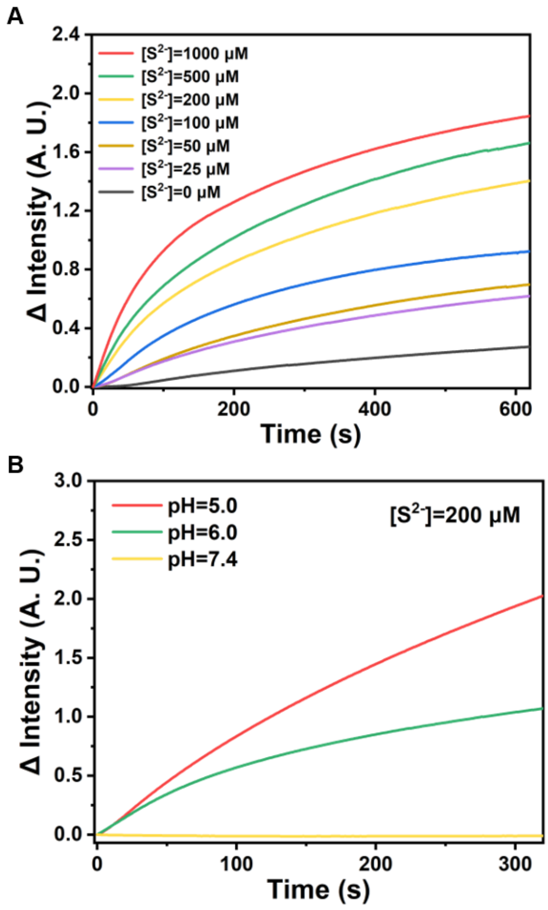

Figure S16. The influence of S^{2-} on LDH catalytic ability. A) The time-course absorbance at 652 nm of LDH in TMB solution (0.8 mM, pH=5.8) after pretreatment with different concentrations of Na_2S (0, 25, 50, 100, 200, 500, 1000 μM). B) The UV-vis absorbance at 652 nm of LDH + Na_2S in TMB solution at different pH values.

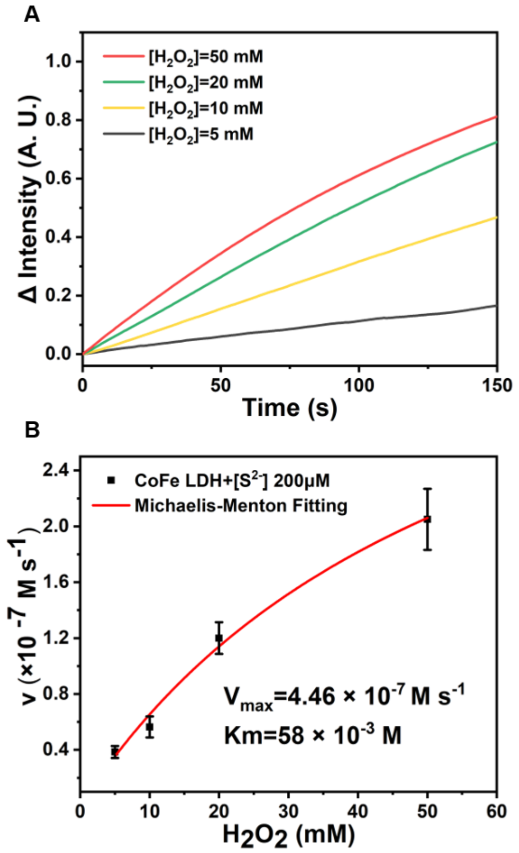

Figure S17. **Catalytic ability of LDH after 200 mM Na_2S pretreatment.** A) The time-course absorbance at 652 nm of LDH in TMB solution with different concentrations of H_2O_2 (5, 10, 20, 50 mM) after 200 mM Na_2S pretreatment and **B)** the corresponding Michaelis–Menten fitting curve of initial •OH generation velocities of LDH against H_2O_2 concentration after 200 mM Na_2S pretreatment.

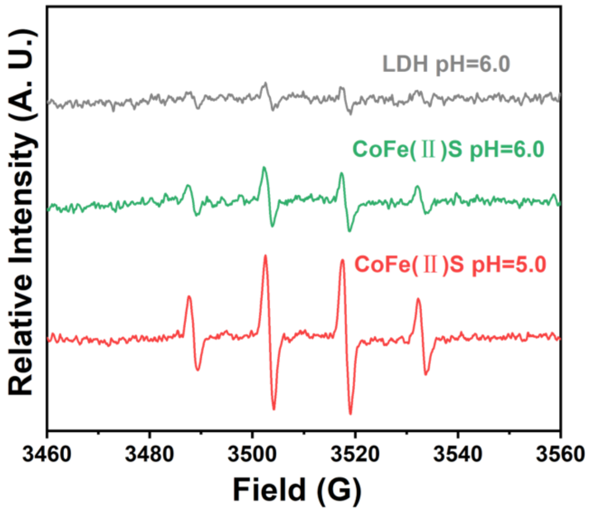

Figure S18. ESR spectra of LDH and CoFe(II)S from the same origin mass of LDH in different conditions.

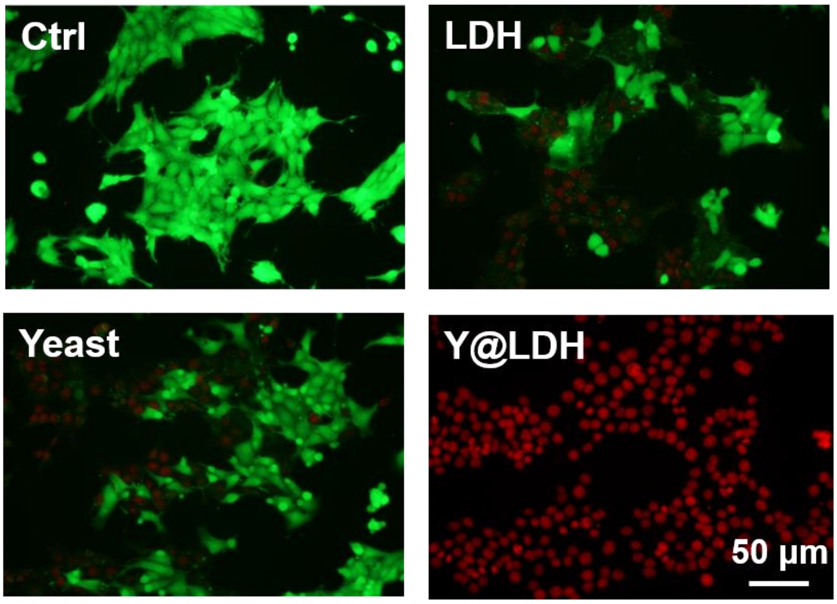

Figure S19. Live/dead staining of 4T1 cells by using calcein-AM/propidium iodide after incubation with different therapeutic agents.

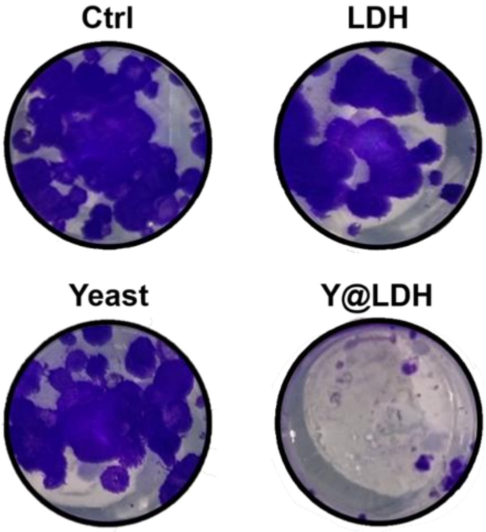

Figure S20. Colony efficiency assay of 4T1 cells after incubation with different therapeutic agents.

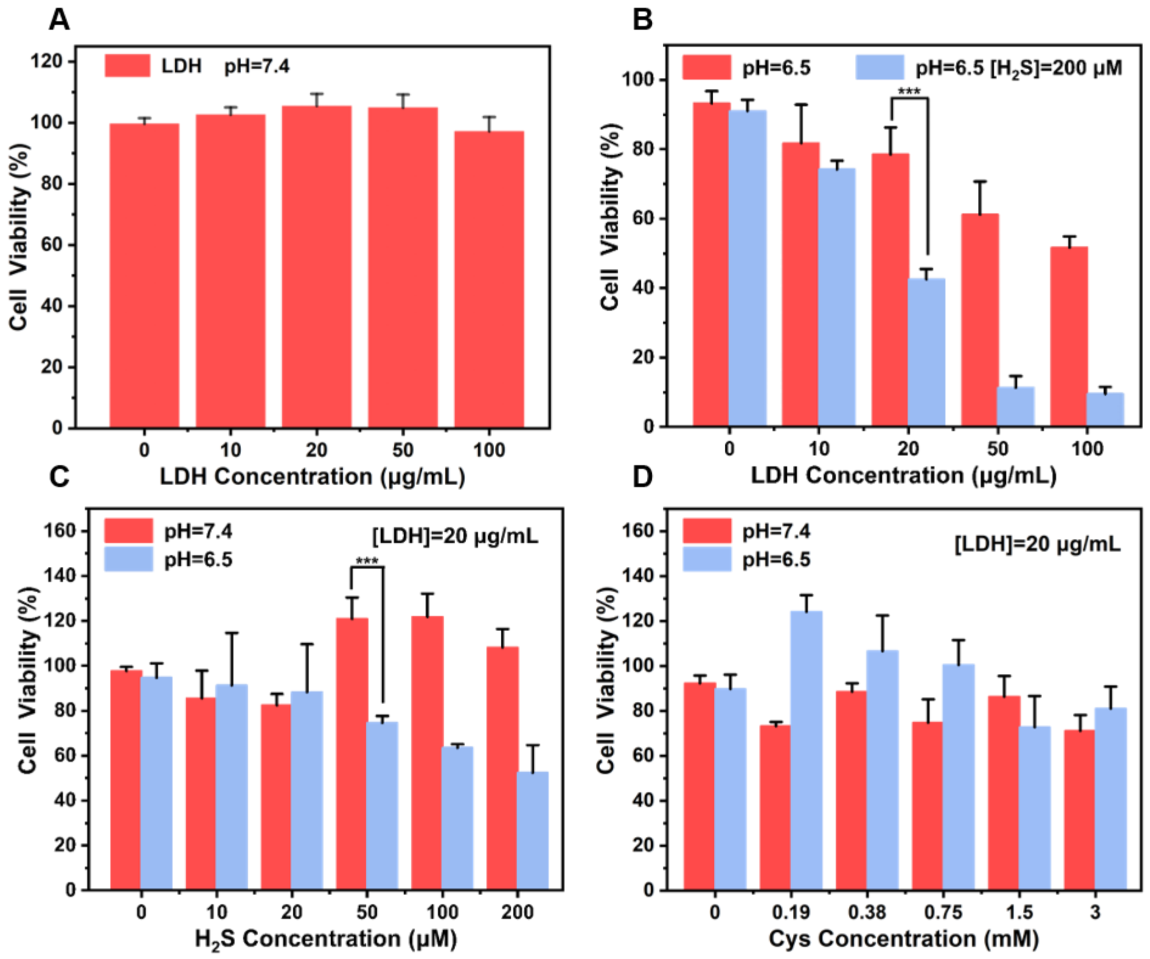

Figure S21. In vitro antitumor properties of LDH. A) Cytotoxicity of LDH to murine mammary carcinoma cells 4T1 in normal culture medium. B) Cytotoxicity of LDH to 4T1 cells at pH 6.5 in the addition of 200 μM H_2S. Cytotoxicity of LDH (20 μg mL^{-1}) in the addition of C) H_2S and D) Cys with different concentrations at pH 7.4 and 6.5.

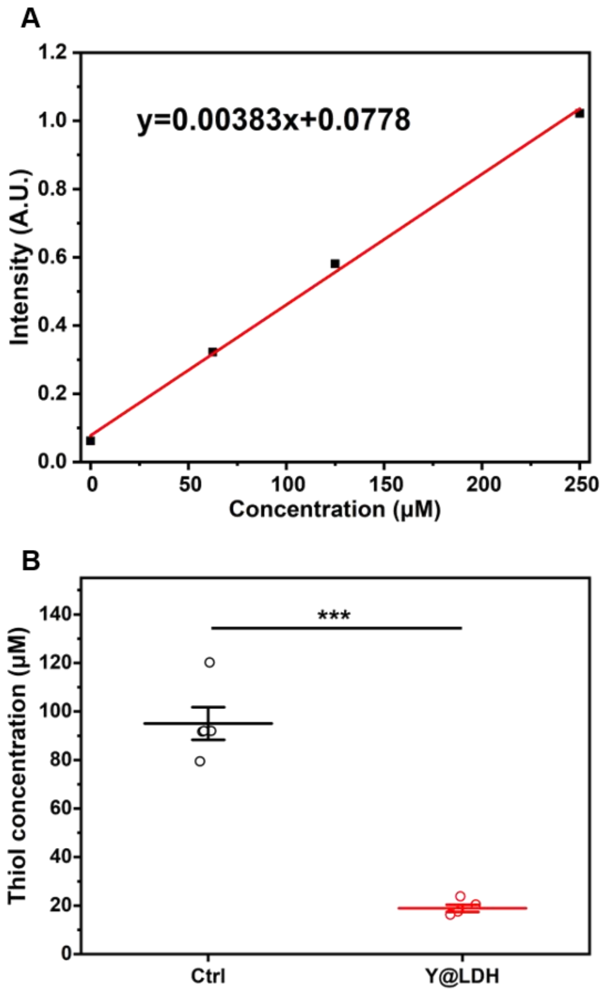

Figure S22. Quantified intracellular thiol concentration. A) GSH standard curve and B) quantified intracellular thiol concentration difference between PBS and Y@LDH treatment.

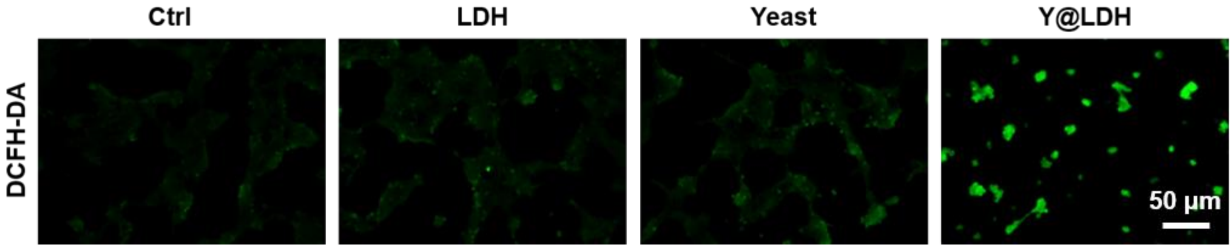

Figure S23. DCFH-DA staining to detect the production of intracellular ROS.

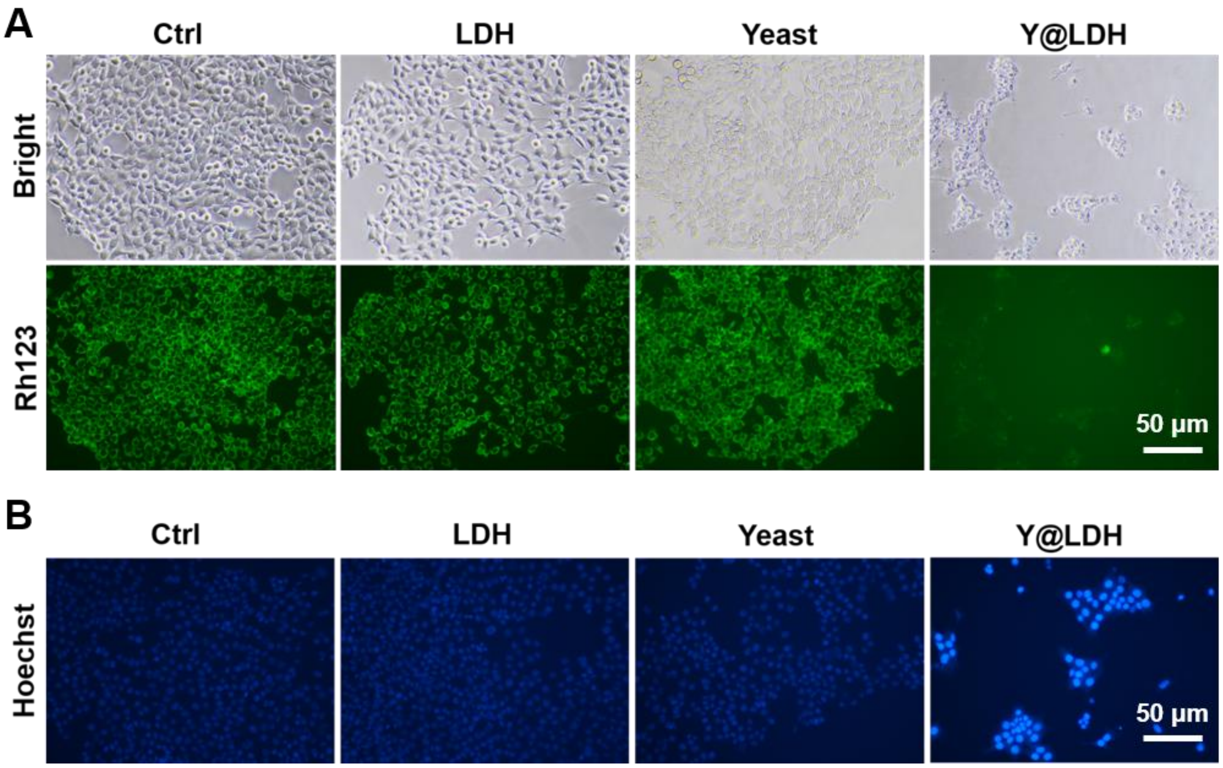

Figure S24. **The influence of Y@LDH on mitochondrial membrane potential and DNA of 4T1 cells in TME-mimic environment.** A) Rh123 staining to indicate mitochondrial membrane potential in cells. B) Hoechst 33324 staining for indicating DNA damage.

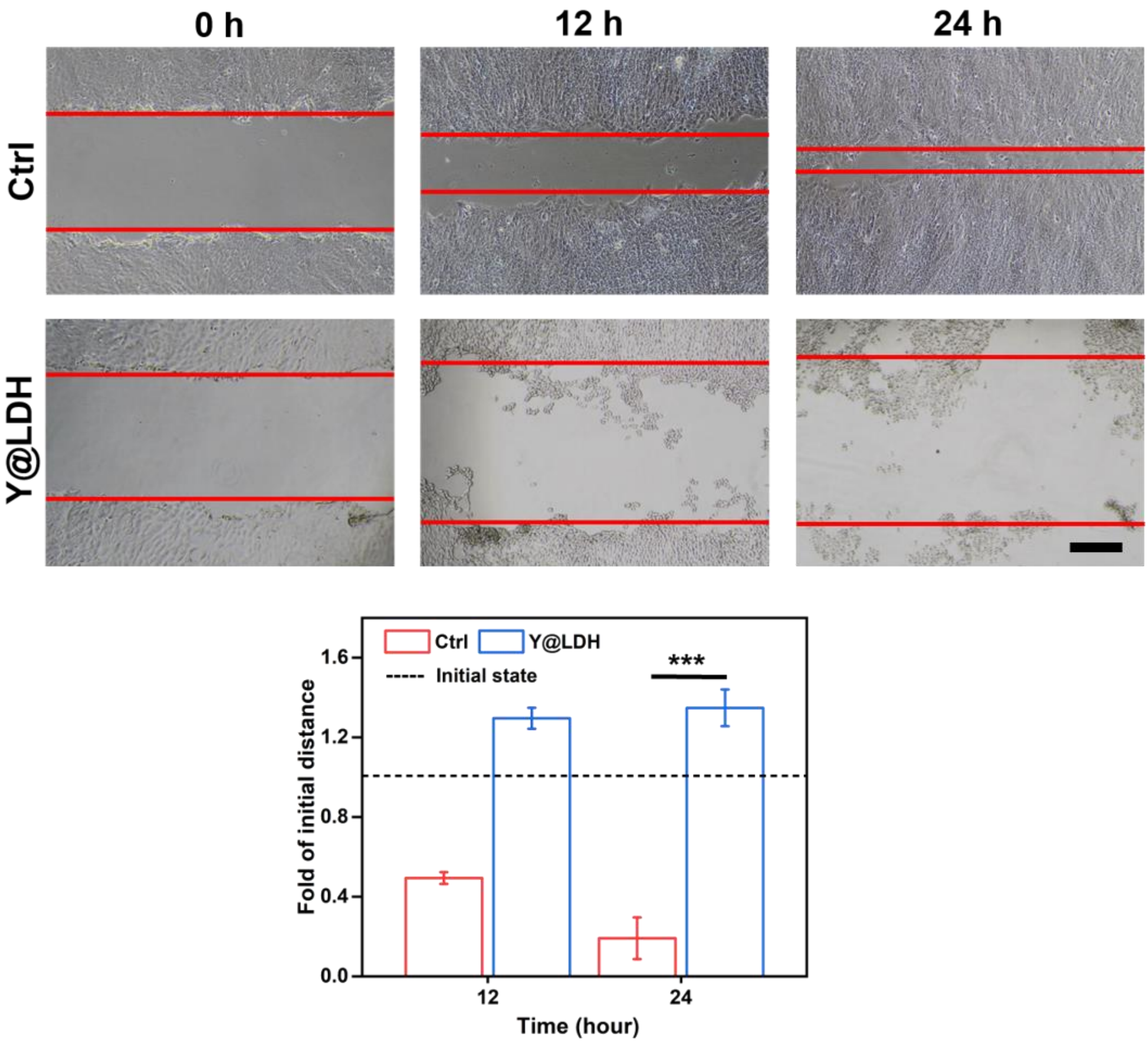

Figure S25. Migration ability of 4T1 cells after treatment with PBS and Y@LDH for 0, 12 and 24 h. Scale bar: 100 µm.

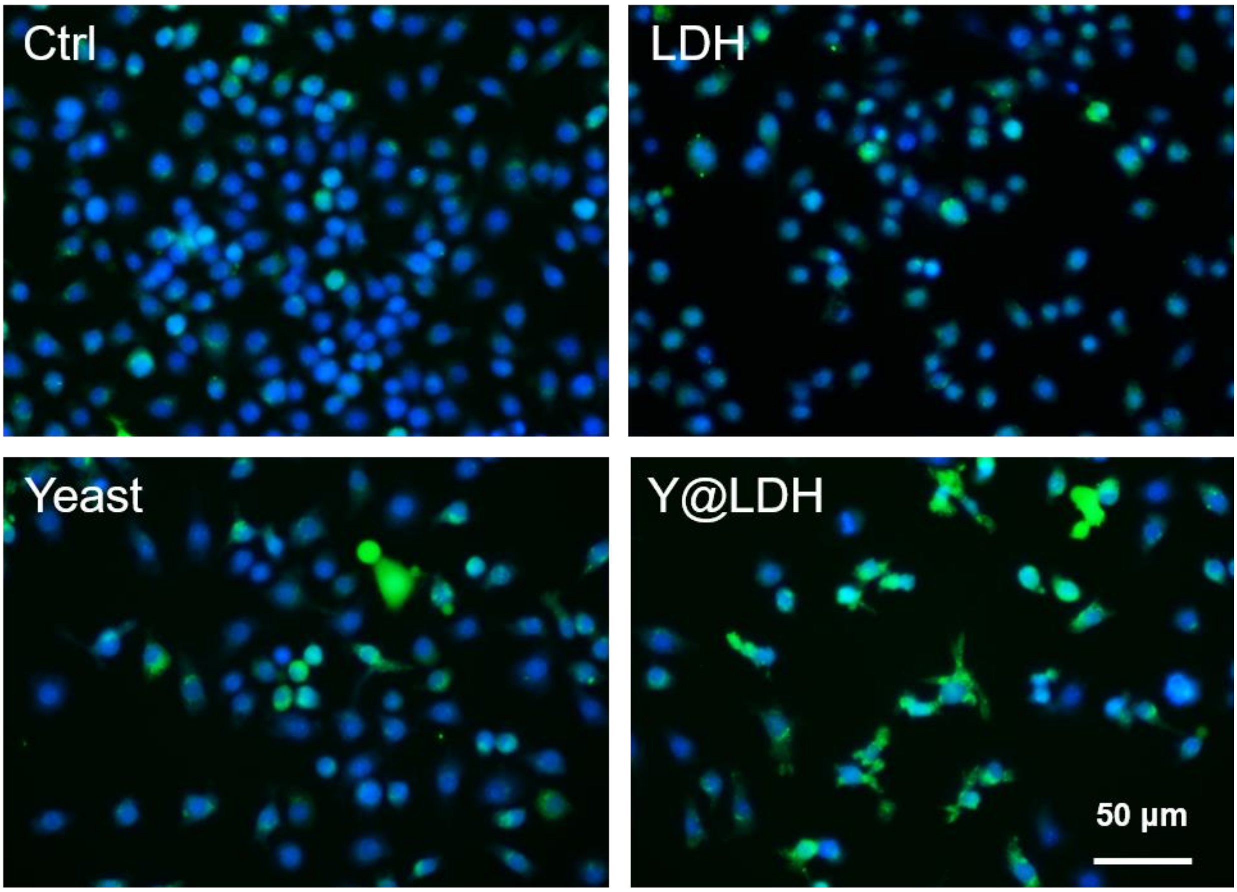

Figure S26. Intracellular NO staining of RAW 264.7 macrophages.

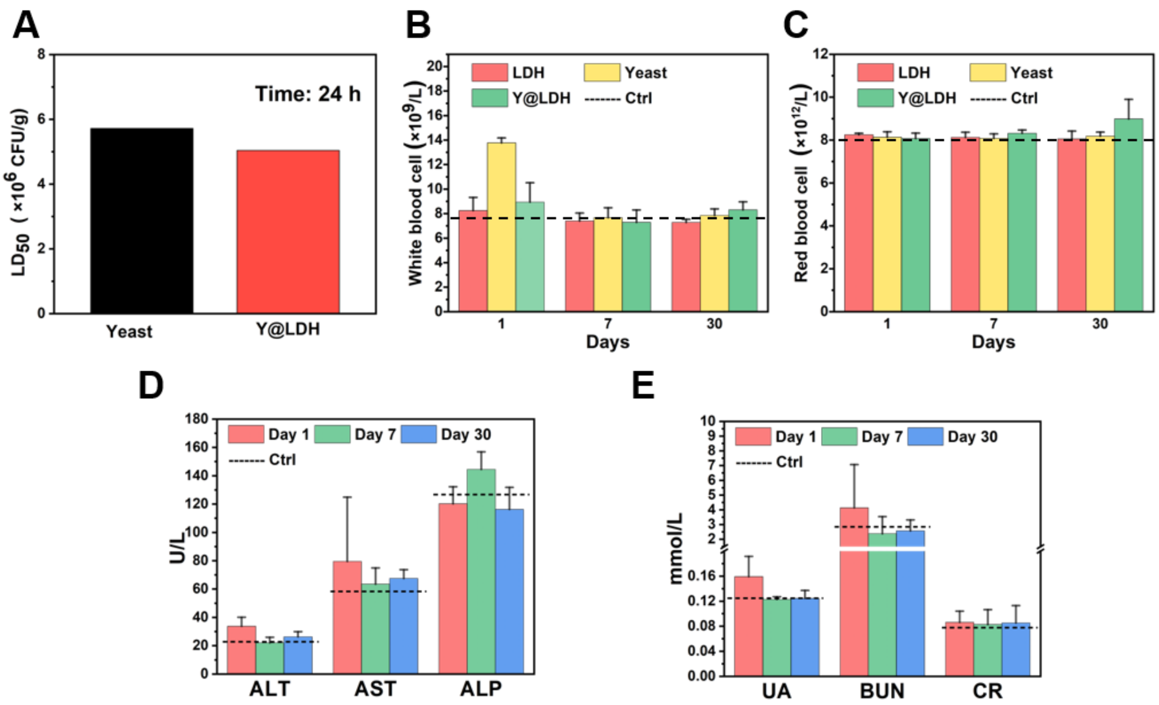

Figure S27. Biosafety of Yeast and Y@LDH. A) Median lethal dose of Yeast and Y@LDH. The variation of B) white blood cell concentration and C) red blood cell concentration at 1st, 7th and 30th day after intravenously injection of Y@LDH. D) Liver function related substrate concentration and E) kidney function related substrate concentration at 1st, 7th and 30th day after intravenously injection of Y@LDH.

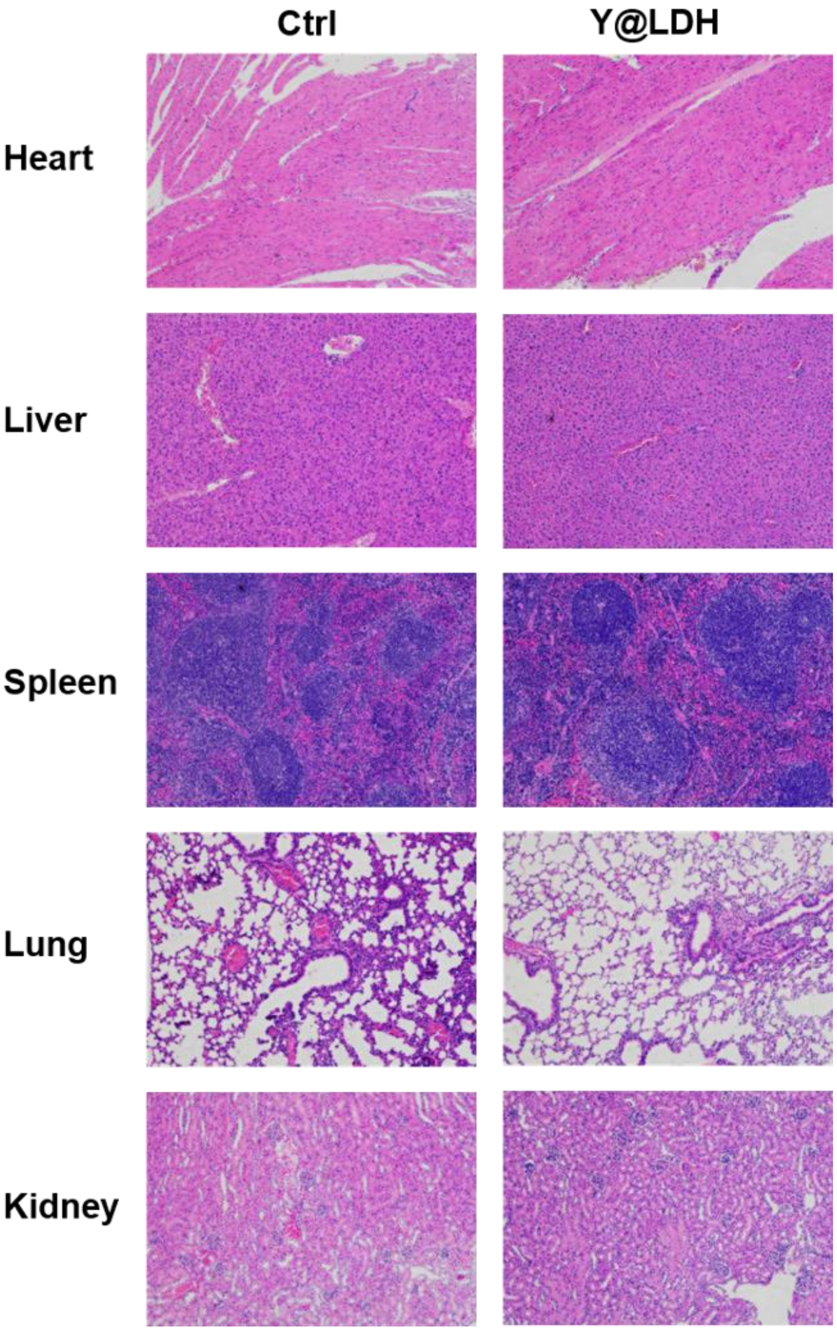

Figure S28. H&E staining images of histologic sections in heart, liver, spleen, lung and kidney after intravenously injecting Y@LDH for 30 days.

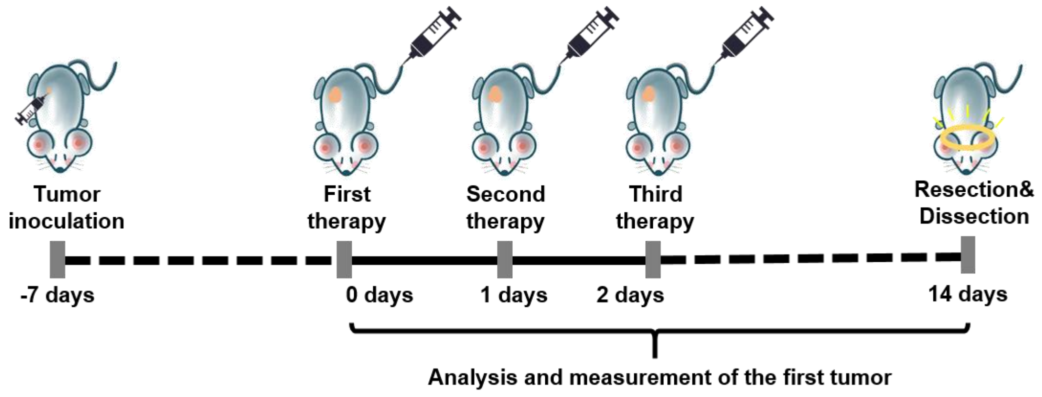

Figure S29. Schematic illustration of therapeutic process on subcutaneous mouse 4T1 tumor.

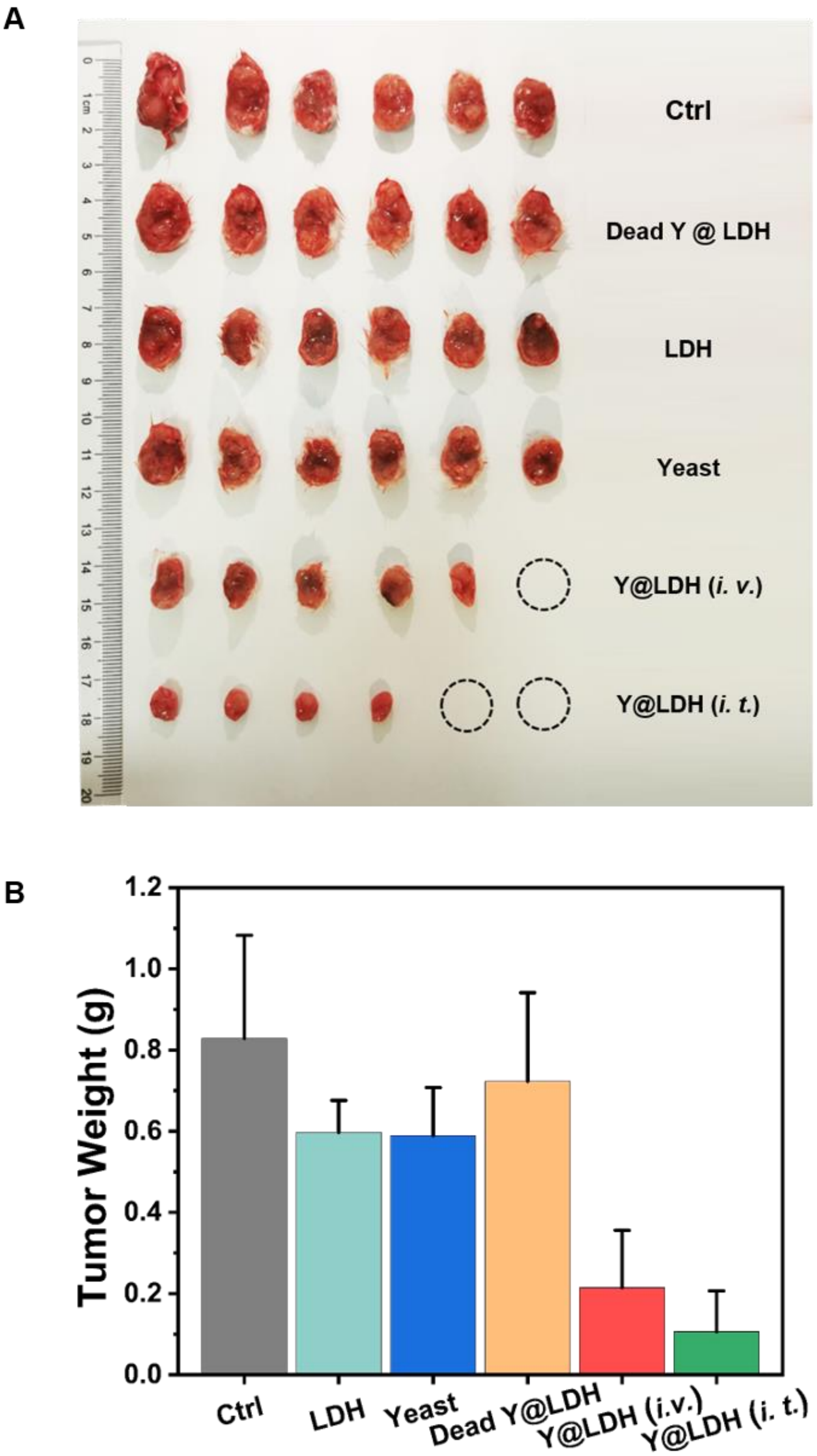

Figure S30. The influence of each therapeutic agent on tumors. A) Photographs of tumors and B) tumor weight on 14th day after different treatments.

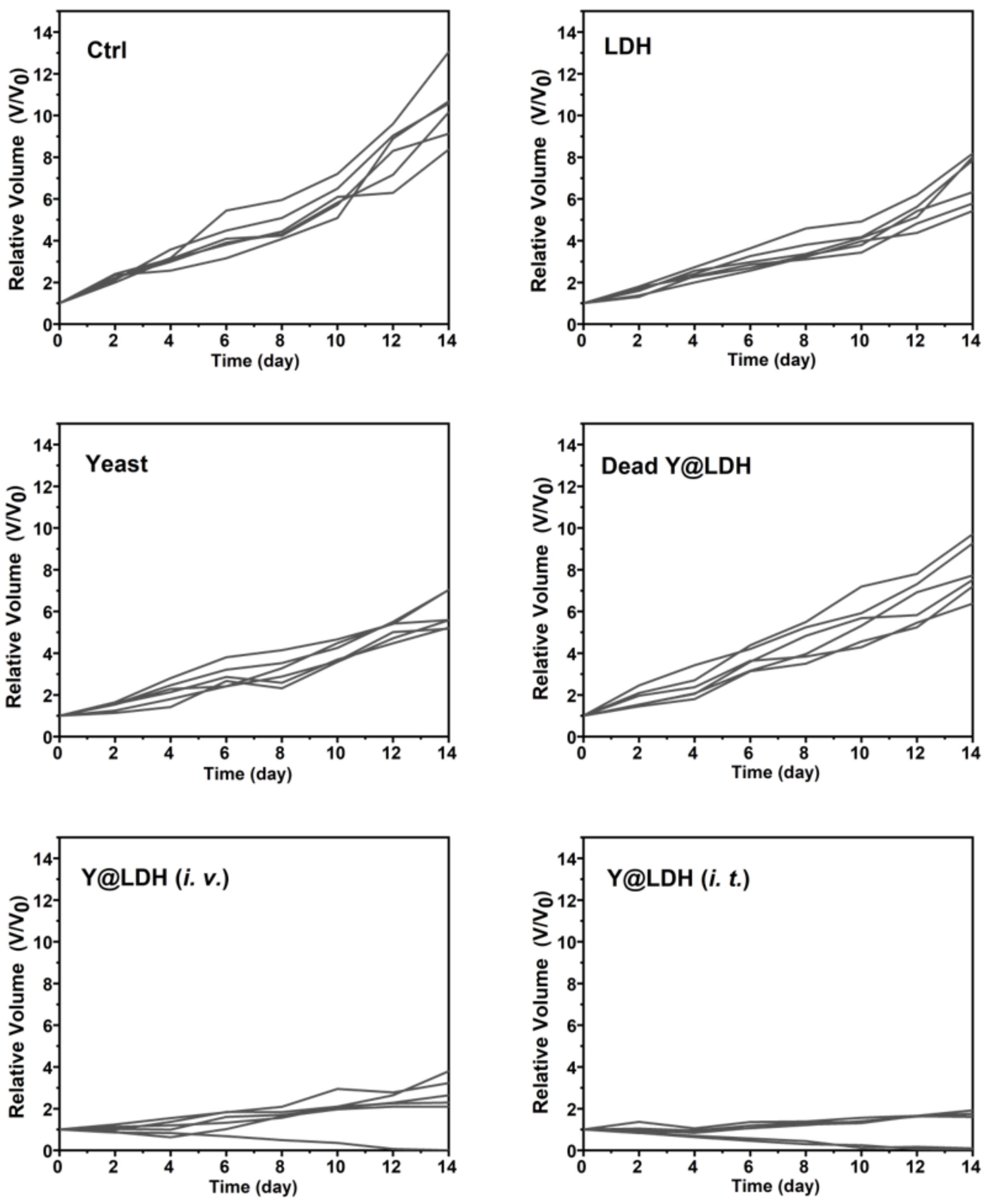

Figure S31. Tumor growth curves of mice in each group.

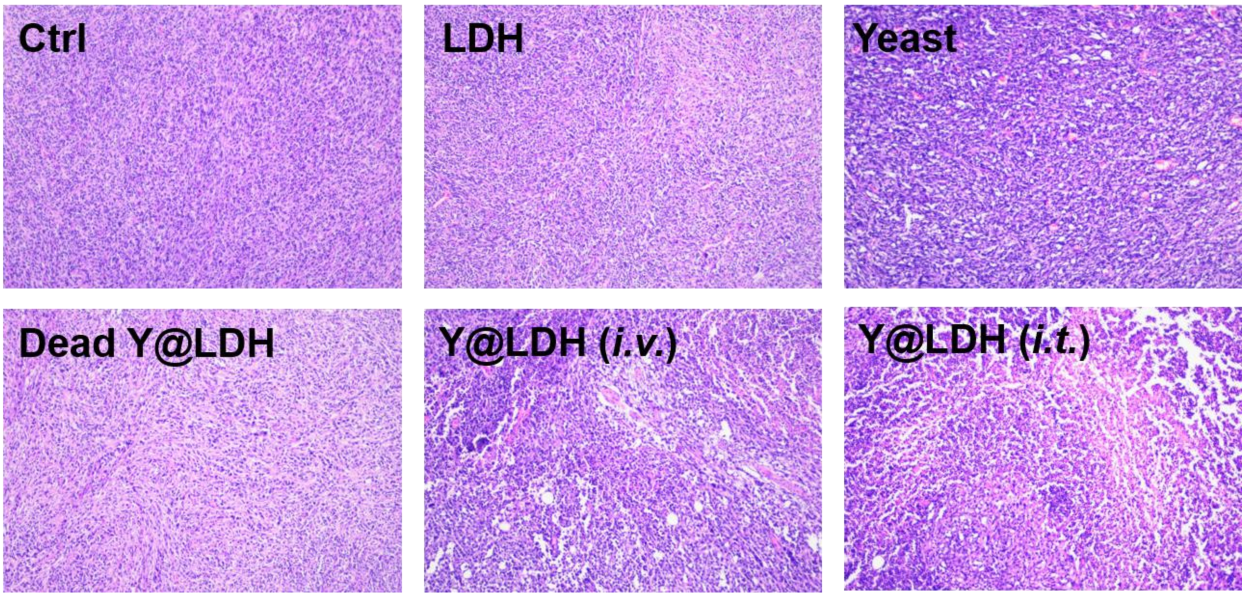

Figure S32. H&E staining of tumor histological sections on 14th day.

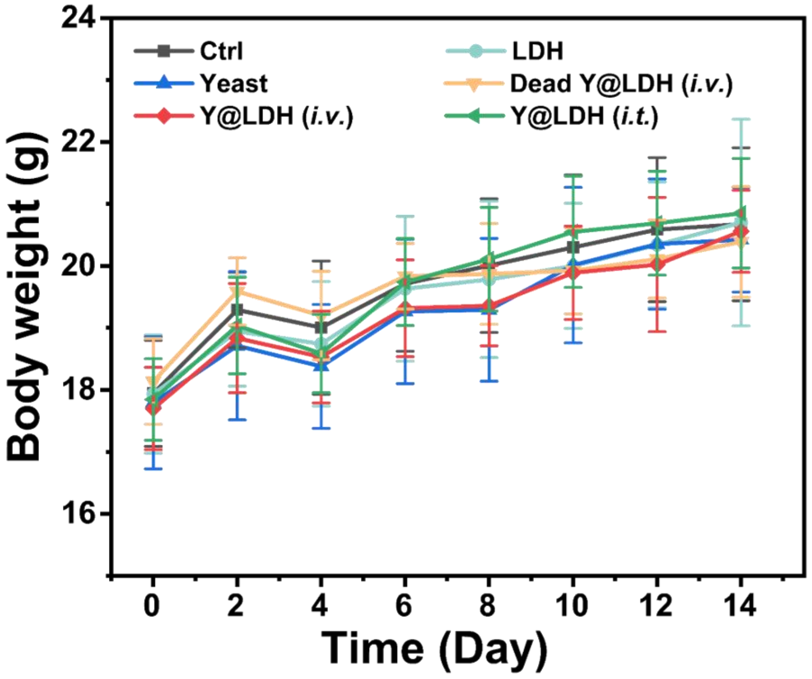

Figure S33. Body weights of mice after injection of different therapeutic agents.

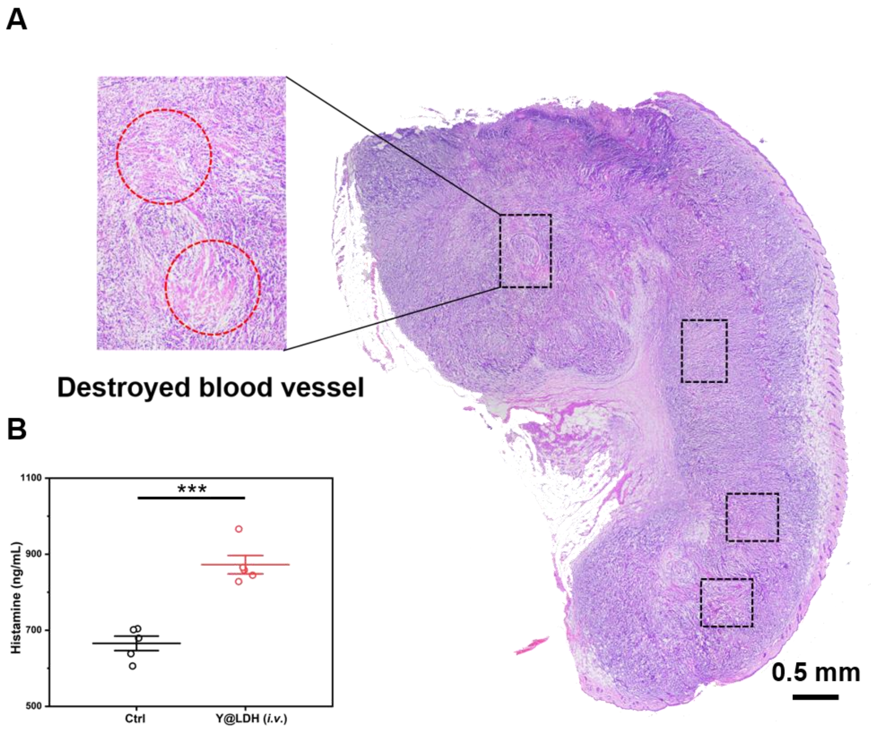

Figure S34. Blood vessel destruction in tumor tissues by Y@LDH. A) H&E staining to exhibit blood destruction ability of Y@LDH. B) Histamine concentrations in blood 1 day after intravenous injection of Y@LDH.

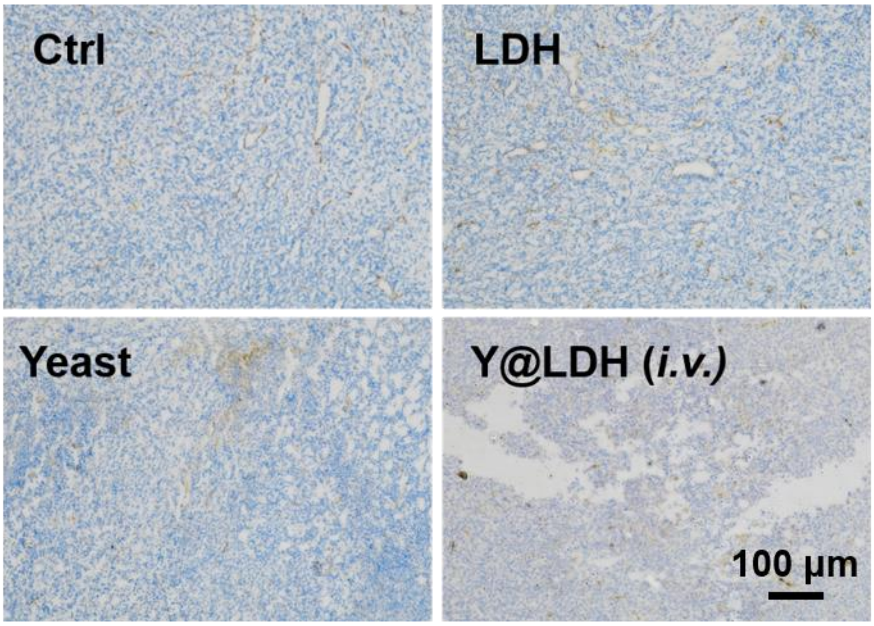

Figure S35. CD31 Immunohistochemistry staining of tumor histological sections after different treatments.

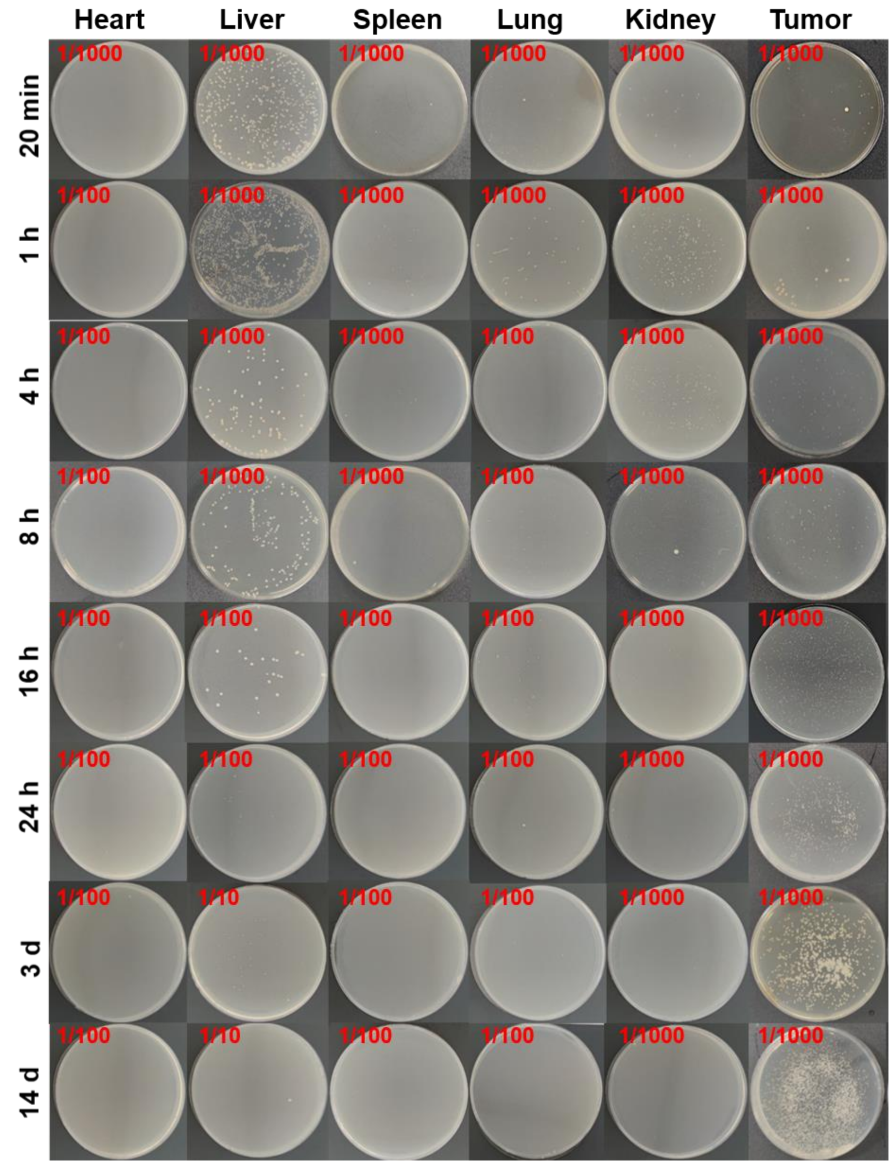

Figure S36. Yeast distribution plating in major organs at different time points after Y@LDH injection.

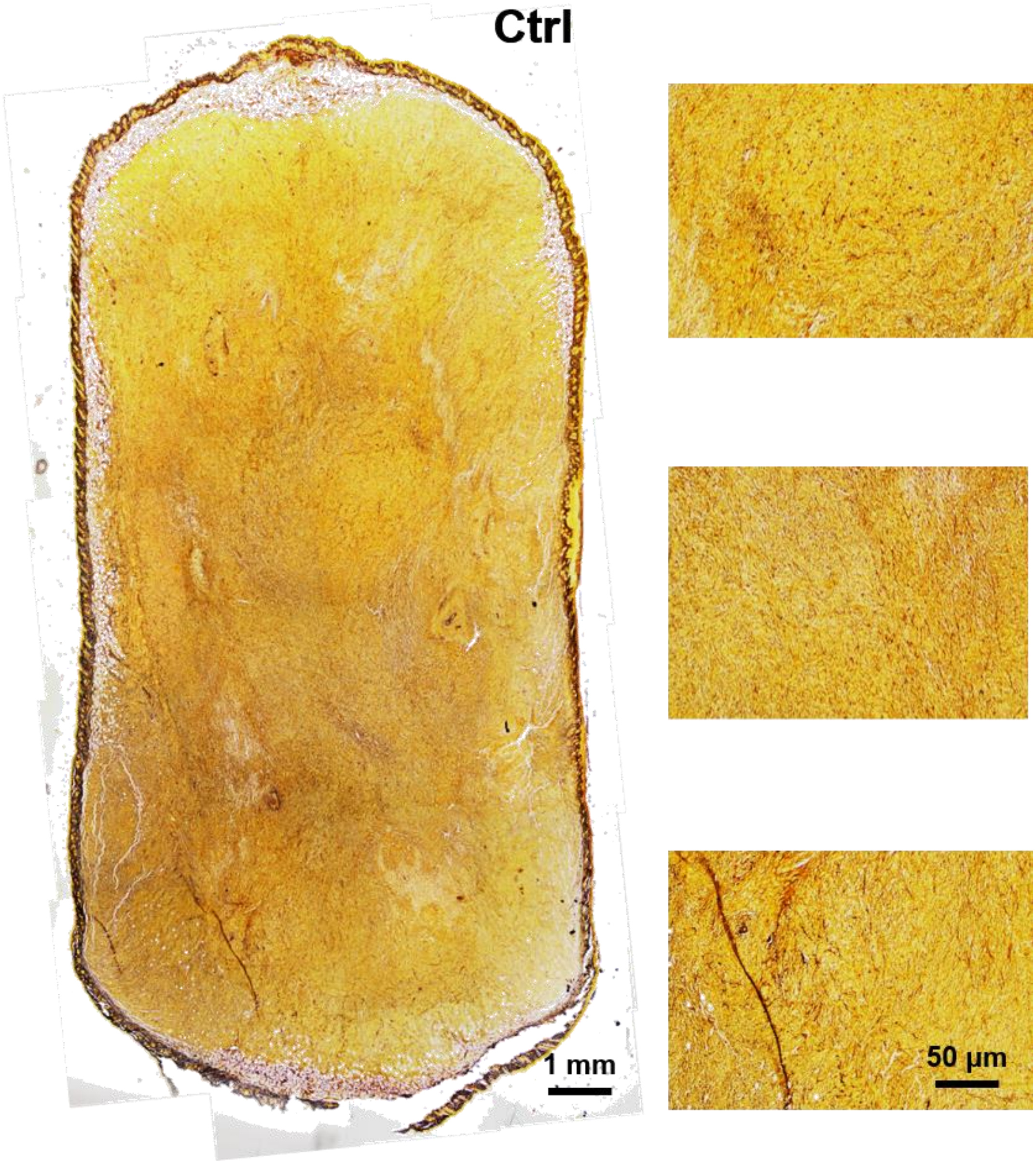

Figure S37. Grocott-Gomori's methenamine silver staining of tumor slice at day 14 after injection of PBS.

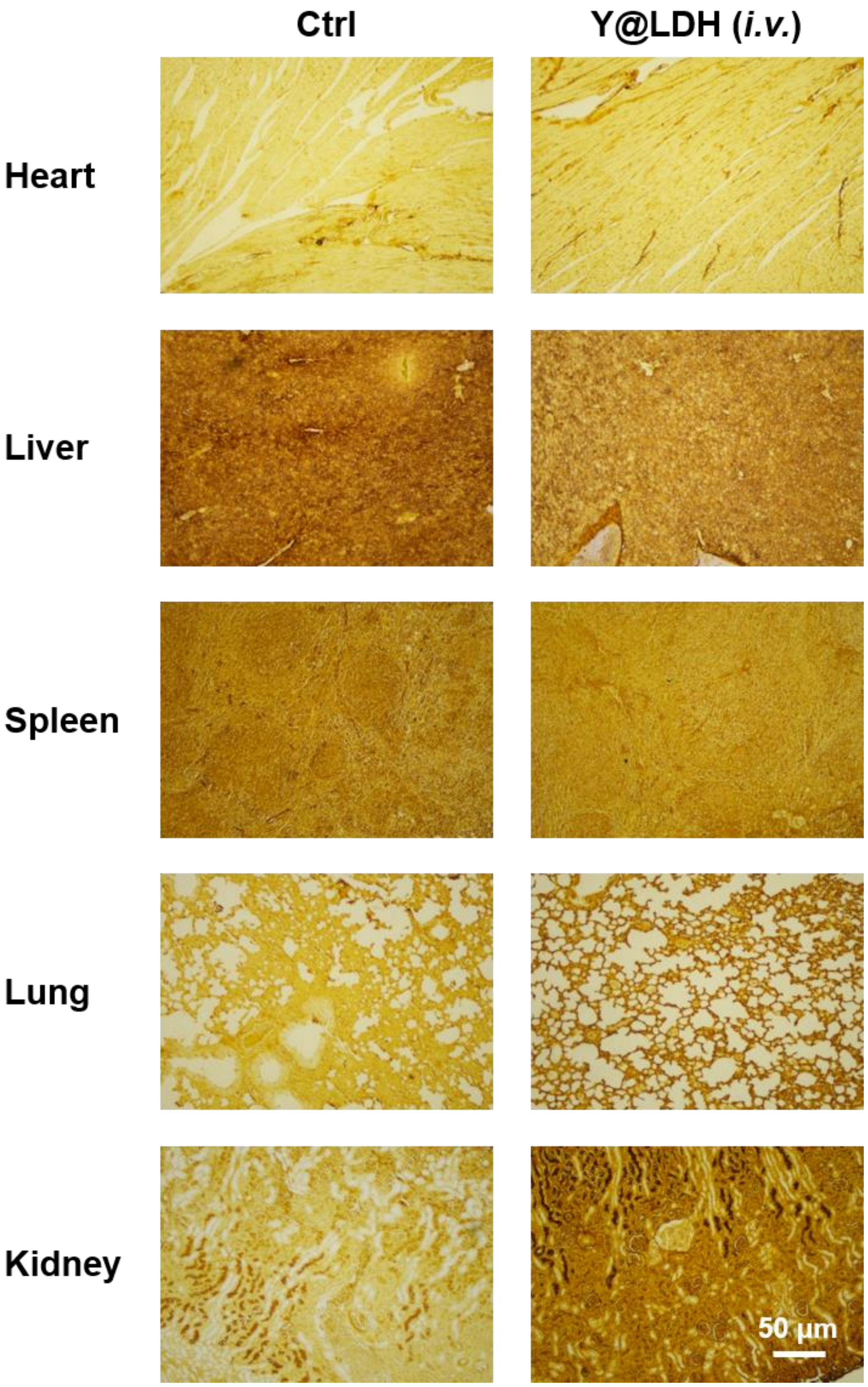

Figure S38. Grocott-Gomori's methenamine silver staining of histological sections in heart, liver, spleen, lung and kidney at day 14 after injection of PBS and Y@LDH.

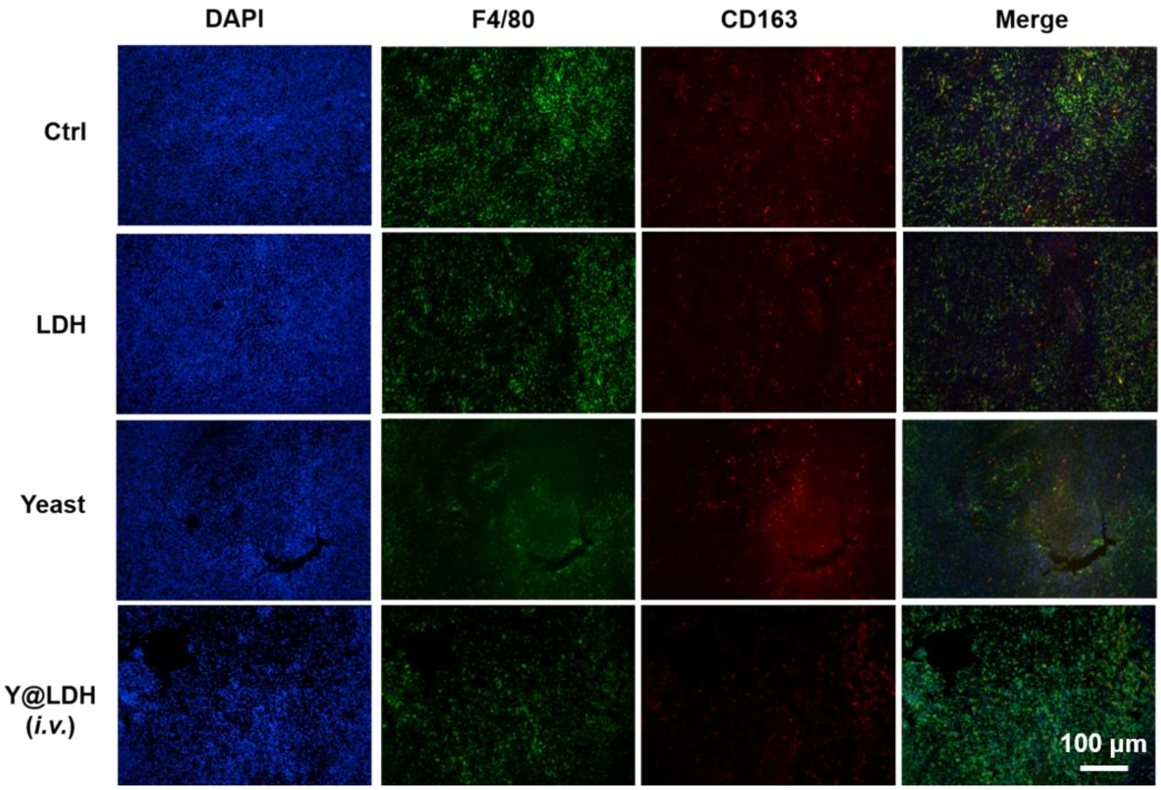

Figure S39. Immunofluorescence staining of F4/80$^+$ CD163$^+$ M2 macrophages in tumor histological sections on 14th day.

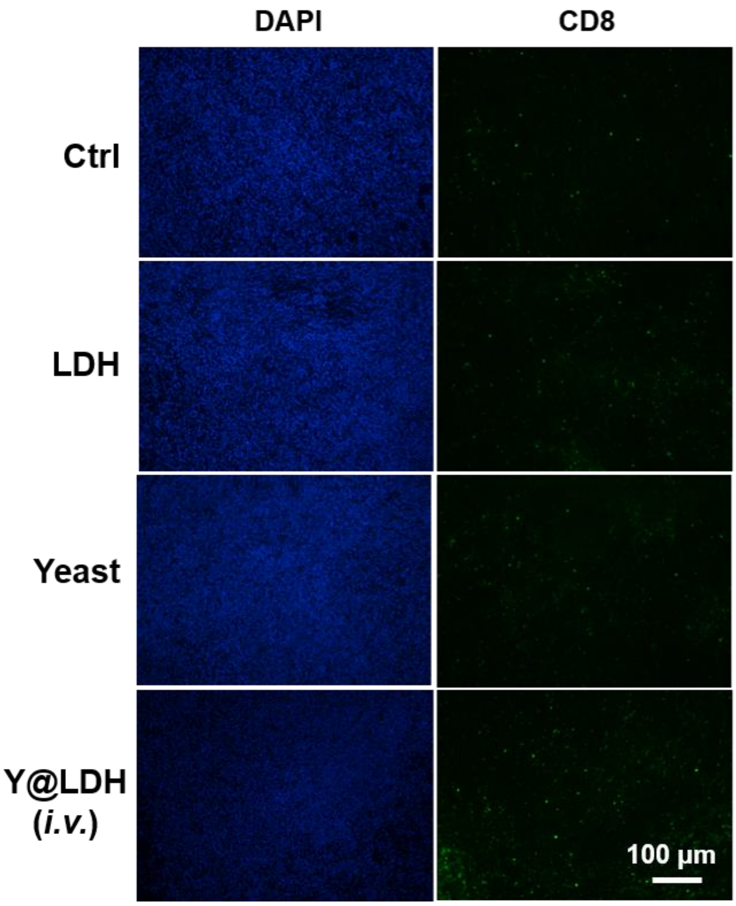

Figure S40. Immunofluorescence staining of CD8^{+} T cells in tumor histologic sections on 14th day.

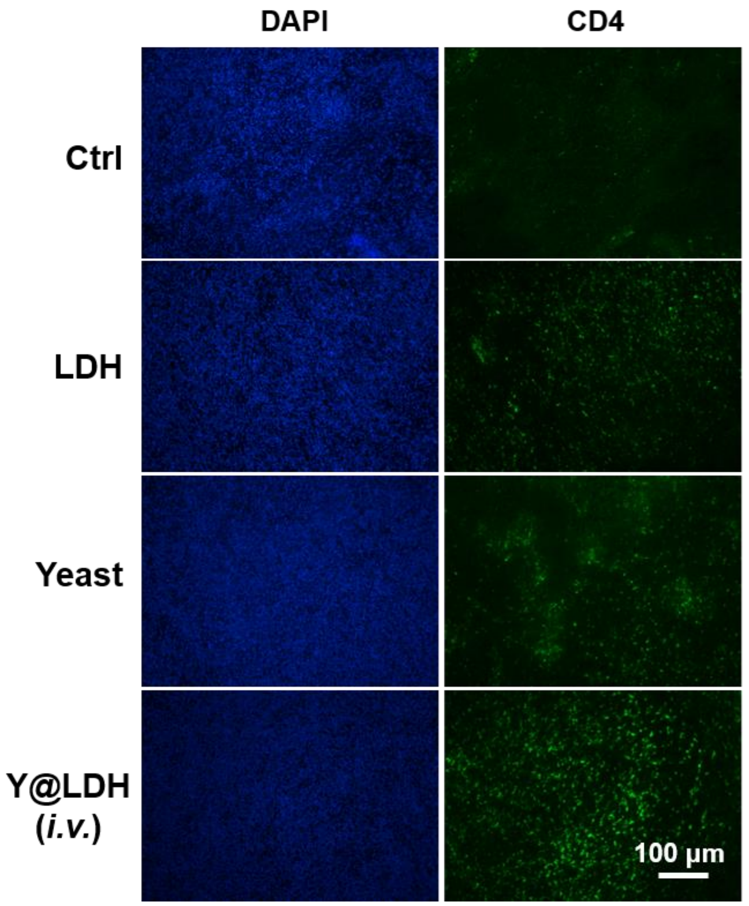

Figure S41. Immunofluorescence staining of $CD4^+$ T cells in tumor histologic sections on 14th day.

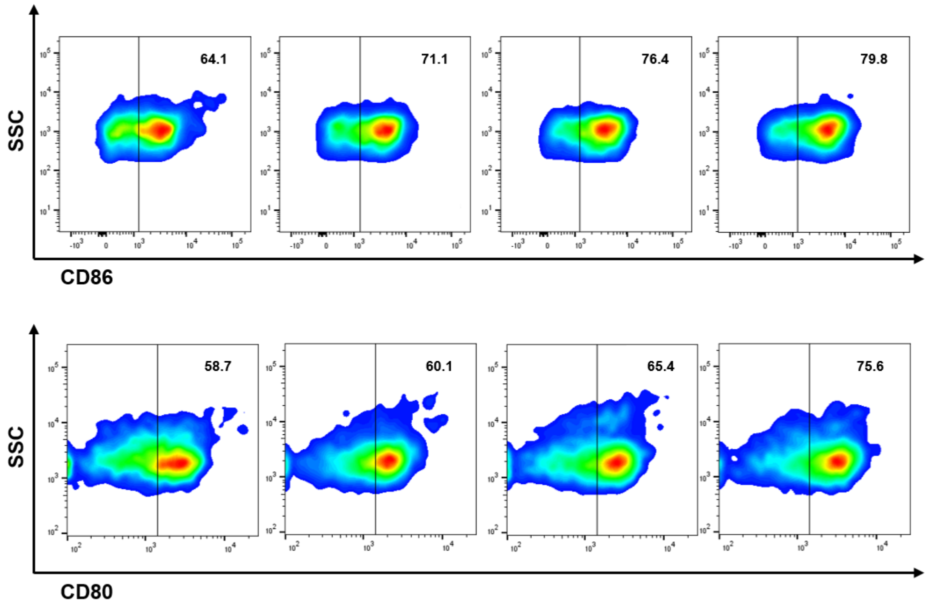

Supporting information Fig. S42. Representative flow cytometry plots illustrating expression of (A) CD86 and (B) CD80 on dendritic cells in draining lymph nodes of tumors 24 h after treatments. Gate: $CD11c^+$ $MHCII^+$

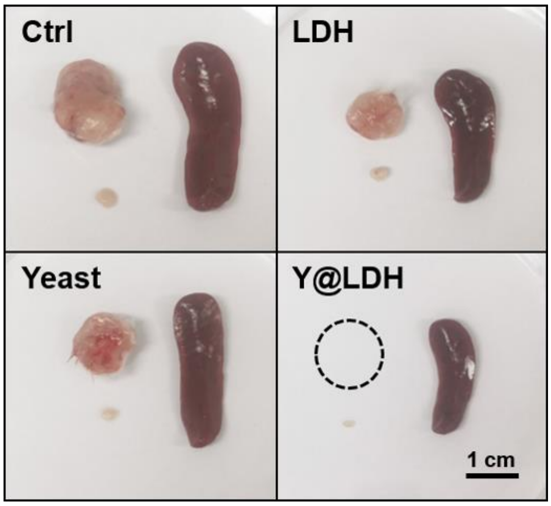

Figure S43. Representative photographs of tumor, spleen and second tumor draining lymph node harvested from mice in different groups 30 days after the rechallenge .

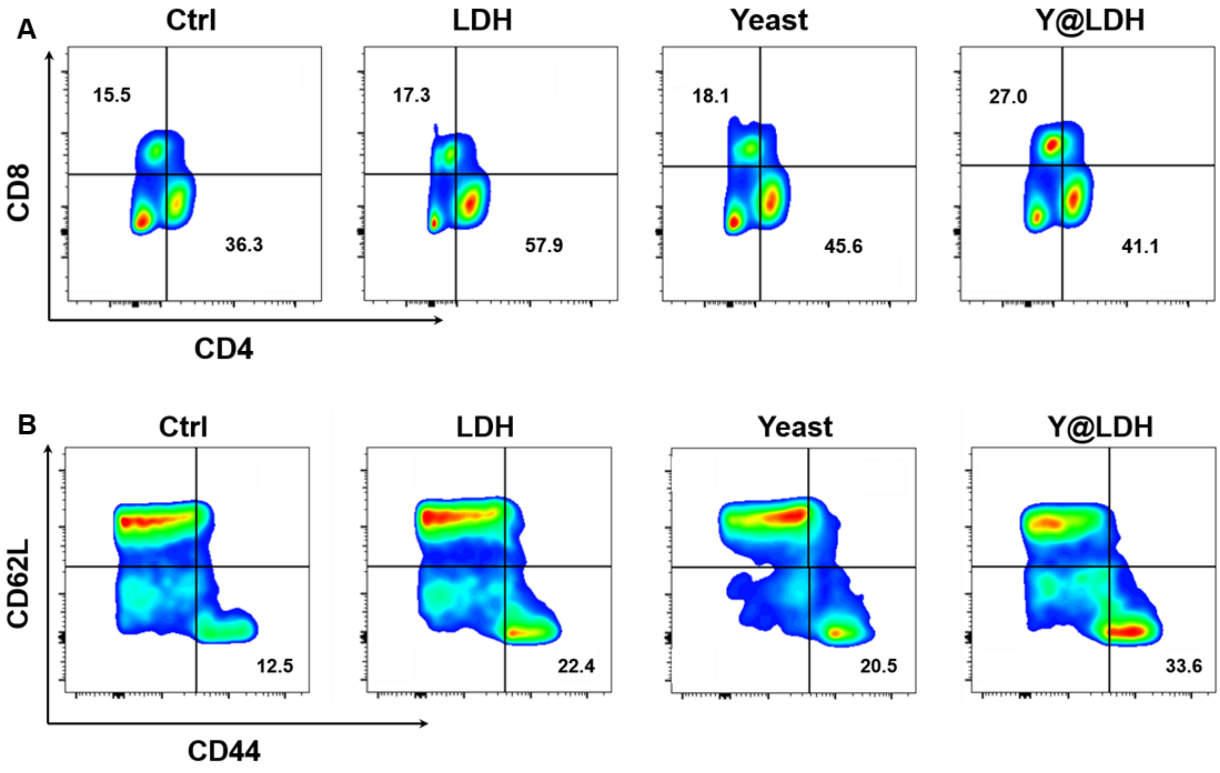

Figure S44. Representative flow cytometry plots showing different groups of leukocytes in draining lymph nodes of the secondary tumor. A) T cells, Gate: $CD45^+$. B) Memory T cells, Gate1: $CD45^+$, Gate2: $CD8^+$.

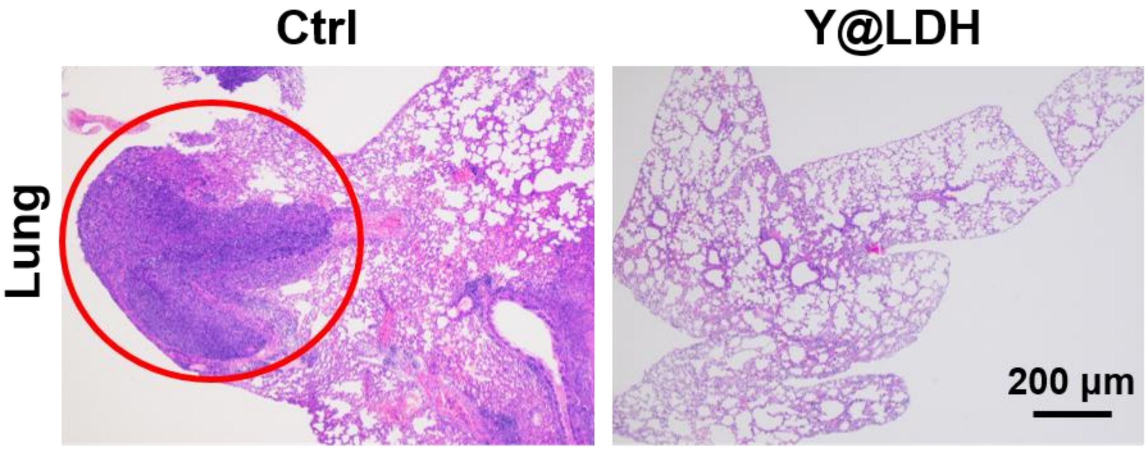

Figure S45. H&E staining of lung histological sections 60 days after treatments of PBS/Y@LDH and surgery.

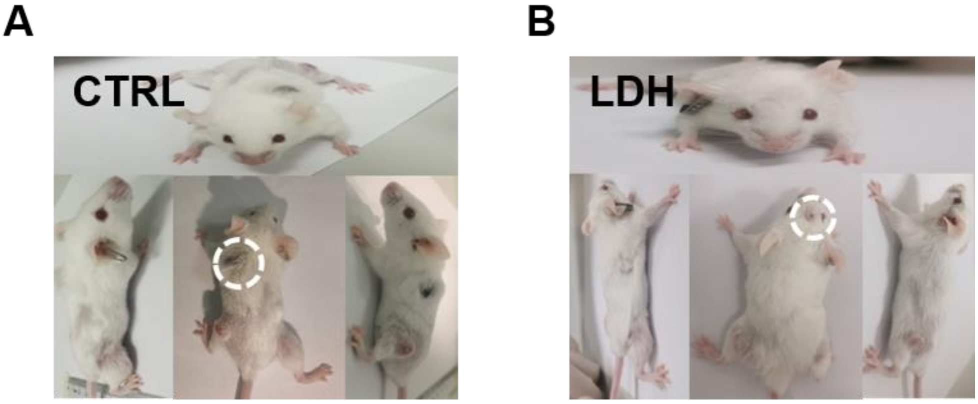

Figure S46. Photographs of bizarre tumor metastasis on mice from A) Control group and B) LDH group.

References

[1] C. Fang, Z. Deng, G. D. Cao, Q. Chu, Y. L. Wu, X. Li, X. S. Peng, G. R. Han, *Adv. Funct. Mater*. **2020**, *30*, 1910085.
